# Focus on PD-1/PD-L1-Targeting Antibodies in Colorectal Cancer: Are There Options Beyond Dostarlimab, Nivolumab, and Pembrolizumab? A Comprehensive Review

**DOI:** 10.3390/molecules30132686

**Published:** 2025-06-21

**Authors:** Mateusz Kciuk, Katarzyna Wanke, Weronika Kruczkowska, Beata Marciniak, Renata Kontek

**Affiliations:** 1 Department of Molecular Biotechnology and Genetics, Faculty of Biology and Environmental Protection, University of Lodz, Banacha Street 12/16, 90-237 Lodz, Poland; katarzyna.wanke@edu.uni.lodz.pl (K.W.); weronika.kruczkowska@stud.umed.lodz.pl (W.K.); beata.marciniak@biol.uni.lodz.pl (B.M.); renata.kontek@biol.uni.lodz.pl (R.K.); 2 Department of Functional Genomics, Medical University of Lodz, 90-752 Lodz, Poland

**Keywords:** antibodies, colorectal cancer, immunotherapy, programmed cell death

## Abstract

The PD-1/PD-L1 pathway has emerged as a critical target in colorectal cancer (CRC) immunotherapy, with pembrolizumab, nivolumab, and dostarlimab demonstrating significant clinical efficacy, particularly in microsatellite instability-high (MSI-H) and mismatch repair-deficient (dMMR) tumors. However, a growing number of additional PD-1/PD-L1 inhibitors, including AMP-224, atezolizumab, avelumab, camrelizumab, durvalumab, envafolimab, sintilimab, spartalizumab, tislelizumab, and toripalimab, are currently under investigation, offering new possibilities for the expansion of treatment options. This review evaluates the therapeutic potential of these emerging agents, assessing their clinical development, mechanisms of action, and potential advantages over established therapies. Additionally, it explores key challenges such as primary and acquired resistance, limited efficacy in microsatellite-stable (MSS) CRC, and the complexities of combination strategies aimed at enhancing immunotherapeutic responses. By addressing these obstacles and highlighting prospects, this review provides insights into the evolving landscape of PD-1/PD-L1-targeted therapies in CRC and their potential to improve patient outcomes.

## 1. Introduction

The incidence of cancer is experiencing a significant and rapid escalation, with 19.3 million cases expected in 2020 and about 10 million deaths attributed to the disease. Colorectal cancer (CRC) is a prevalent form of cancer, ranking third in terms of frequency and second in terms of cancer-related deaths globally. Despite ongoing advancements in the comprehension of cancer development and evolution, a notable deficit in efficacious therapy options persists for numerous forms of the disease [1,2].

Classical chemotherapy is generally accepted as the core component of cancer treatment. Chemotherapeutic agents target rapidly dividing cells, aiming to disrupt processes such as DNA replication, mitosis, and metabolic pathways critical for cell proliferation. For almost two decades, the chemotherapy-based CRC treatment was mainly focused on various combinations of four drugs, 5-fluorouracil (5FU), irinotecan, oxaliplatin, and leucovorin [3]. However, the administration of this combination may result in a range of adverse effects, as it might impact the proliferation of healthy cells in tissues such as the bone marrow and the digestive tract [4,5].

The introduction of targeted therapies has represented a significant advancement in the treatment of CRC. Unlike conventional chemotherapy, which indiscriminately affects both cancerous and normal cells, targeted therapies are designed to specifically disrupt molecular pathways and processes critical to cancer cell survival and proliferation. By focusing on tumor-specific mechanisms, such as signaling pathways, growth factor receptors, or genetic mutations, these therapies aim to minimize damage to healthy tissues [6,7,8,9]. The targeted treatment strategy for CRC involves the use of monoclonal antibodies (mAbs) and small-molecule inhibitors. The mAbs are molecules that can bind to specific proteins on the surface of cancer cells. In CRC, there are two common targets, as described in the following.

Epidermal growth factor receptor (EGFR): mAbs such as cetuximab [10] and panitumumab [10] can block the EGFR, which is involved in the cell proliferation, growth, and metastasis of cancer cells [11]. The efficacy of EGFR mAbs has been assessed in the context of first, second, and third-line therapies. These mAbs have been investigated both as standalone treatments and in conjunction with other chemotherapeutic drugs. Both antibodies have demonstrated efficacy in reducing the likelihood of tumor progression and enhancing overall survival (OS), progression-free survival (PFS), and quality of life (QoL) in patients with refractory CRC [12,13,14,15,16].

Vascular endothelial growth factor (VEGF): bevacizumab and ramucirumab represent examples of mAbs that target VEGF, a protein that stimulates the formation of new blood vessels in tumors upon binding with its receptor. By blocking VEGF, these agents can reduce the blood supply to the tumor, starving it of nutrients and oxygen [17,18,19,20,21].

Regorafenib is a multi-kinase inhibitor that has demonstrated significant efficacy in the treatment of metastatic colorectal cancer (mCRC), particularly in patients who have progressed on standard therapies. It targets multiple signaling pathways implicated in tumor proliferation, angiogenesis, and the tumor microenvironment, including vascular endothelial growth factor receptors (VEGFR1-3), platelet-derived growth factor receptor (PDGFR), fibroblast growth factor receptor (FGFR), and oncogenic kinases such as KIT and RET. Clinical trials, such as the CORRECT (NCT01103323) and CONCUR (NCT01584830) studies, have shown that regorafenib improves OS and PFS in heavily pretreated mCRC patients, with a manageable safety profile. The drug’s anti-angiogenic and anti-proliferative effects disrupt tumor growth and progression by inhibiting vascularization and reducing tumor cell viability [22,23,24].

Furthermore, larotrectinib, also known as BAY2757556, is an orally accessible pyrazolo[1,5-a]pyrimidine small-molecule inhibitor that received approval in 2018 for the treatment of solid tumors that possess neurotrophic tropomyosin receptor kinases (NTRK) gene fusion and for which satisfactory alternative therapy options are lacking or in which there has been disease progression after treatment [25,26]. Gene fusions, including NTRK rearrangements, are infrequent occurrences that have the potential to serve as a novel therapeutic target for the enhancement of the efficacy of cancer treatment in individuals with CRC. The efficacy and safety of larotrectinib and entrectinib (first-generation tropomyosin kinase inhibitors approved in 2019) have been confirmed in mCRC cancer patients with NTRK pathogenic fusions. Moreover, there is a growing emergence of second-generation compounds that can effectively counteract the acquired resistance to NTRK inhibition [27,28]. The primary obstacles lie in the effective execution of the screening process for *NTRK* fusions within the broader oncological community and in integrating larotrectinib into existing therapy algorithms [26,27]. Additionally, in 2020 the FDA approved encorafenib in combination with cetuximab for adult patients with mCRC harboring serine/threonine-protein kinase B-Raf *BRAF V600E* mutation after their previous therapy [29].

Programmed cell death protein 1 (PD-1) and its ligand PD-L1 have emerged as critical targets in immunotherapy for CRC, particularly in the context of microsatellite instability-high (MSI-H) or mismatch repair-deficient (dMMR) tumors. These tumors are characterized by a high tumor mutational burden (TMB), leading to increased neoantigen presentation and heightened immune responsiveness. PD-1/PD-L1 inhibitors, such as pembrolizumab, nivolumab, and dostarlimab, block the immune checkpoint interaction between PD-1 on T cells and PD-L1 on tumor cells, restoring T-cell activity and enabling anti-tumor immune responses. Clinical trials have demonstrated the remarkable efficacy of PD-1/PD-L1 inhibitors in MSI-H/dMMR CRC, with durable responses and improved overall survival compared to standard chemotherapy. However, the benefit of these therapies in microsatellite-stable (MSS) CRC, which constitutes the majority of CRC cases, remains limited due to lower immunogenicity and a less inflamed tumor microenvironment [30].

While pembrolizumab, nivolumab, and dostarlimab were thoroughly examined in the context of CRC treatment, there are other PD-1/PD-L1 pathway targeting antibodies, such as AMP-224, atezolizumab, avelumab, camrelizumab, durvalumab, envafolimab, sintilimab, spartalizumab, tislelizumab, and toripalimab, at various stages of development. This review aims to evaluate their therapeutic potential in this context and describe obstacles to and prospects for their clinical use.

## 2. PD-1/PD-L1 Checkpoint

**Figure 1:**
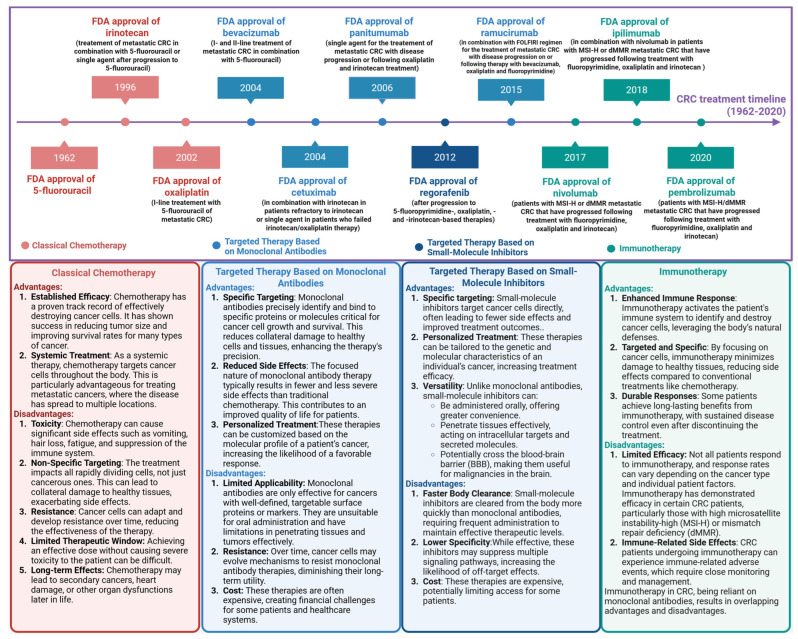
The timeline (1962–2020) of CRC treatment includes classical chemotherapeutic agents (red), targeted therapies with the use of monoclonal antibodies (light blue), small-molecule inhibitors (dark blue), and immunotherapeutic agents (dark cyan). Each treatment approach has distinct characteristics that need to be carefully assessed before their clinical implementation (box below the timeline). Based on [31,32]. Created in BioRender. Kciuk, M. (2025) https://BioRender.com/qosmip2 (accessed on 20 June 2025).

The evolution of CRC treatment, transitioning from traditional modalities such as surgery, chemotherapy, and radiation therapy to contemporary approaches like targeted therapies and immunotherapy, underscore the substantial advancements achieved in this field. Understanding the historical progression of CRC therapies not only highlights their real-world impact but also provides insights into current practices and informs future therapeutic innovations. For clinicians and healthcare providers, an awareness of this historical context is instrumental in making informed treatment decisions tailored to individual patient needs. Each therapeutic modality is associated with distinct advantages and limitations that must be carefully evaluated to optimize patient outcomes. Figure 1 illustrates the timeline of CRC treatment development spanning from 1962 to 2020 [31,32].

PD-1 (CD279) is a type I transmembrane protein expressed on activated T cells, B cells, and NK cells, acting as a co-inhibitory receptor to suppress excessive immune activation. Its interaction with ligands PD-L1 (CD274) and PD-L2, expressed on tumor cells and immune cells within the tumor microenvironment (TME), attenuates T-cell receptor (TCR) signaling and co-stimulatory pathways (e.g., CD28), promoting immune escape [33,34].

PD-L1 expression in tumors is regulated by inflammatory cytokines (e.g., interferon gamma (IFN-γ)) [35], hypoxia-inducible factors (HIFs) [36], genomic alterations [37,38,39,40], and epigenetic modifications [40,41,42,43].

**Figure 2:**
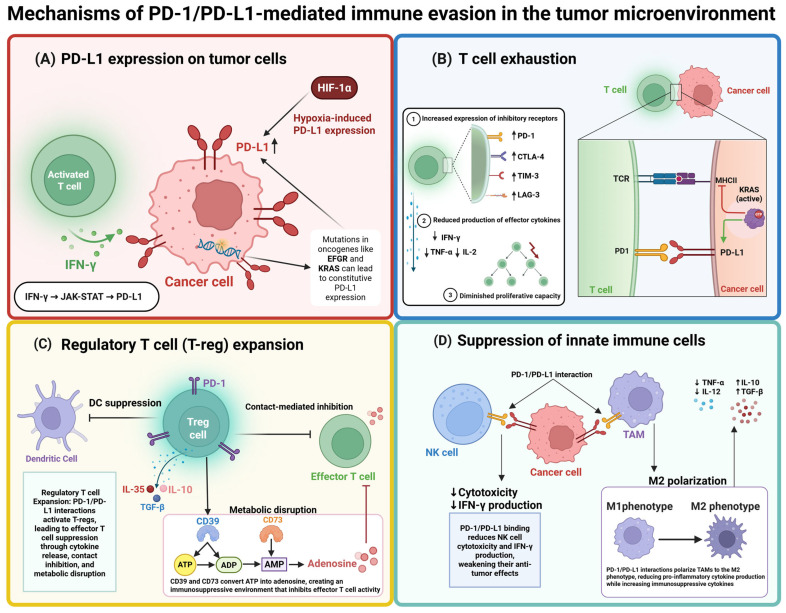
Mechanisms of programmed cell death protein 1/programmed death-ligand 1 (PD-1/PD-L1)-mediated immune evasion in the tumor microenvironment. This illustrates the complex interactions between tumor cells and various immune cells in the context of PD-1/PD-L1-mediated immune evasion. The diagram is divided into four main sections (**A**–**D**), each highlighting a key aspect of this process. (**A**) **PD-L1 expression on tumor cells:** Tumor cells upregulate PD-L1 expression in response to various stimuli. Interferon gamma (IFN-γ) produced by activated T cells induces PD-L1 expression through the Janus kinase–signal transducer and activator of transcription (JAK-STAT) signaling pathway. Hypoxia in the tumor microenvironment (TME) activates hypoxia–inducible factor 1 alpha (HIF-1α), which directly increases PD-L1 expression. Genomic instability in cancer cells, including mutations in oncogenes like epidermal growth factor receptor (EGFR) or Kirsten rat sarcoma viral oncogene homolog (KRAS), can also lead to constitutive PD-L1 expression. (**B**) **T cell exhaustion:** Chronic exposure of T cells to PD-L1-expressing tumor cells leads to T-cell exhaustion. Exhausted T cells are characterized by increased expression of inhibitory receptors (PD-1, cytotoxic T-lymphocyte-associated protein 4 (CTLA-4), T-cell immunoglobulin and mucin-domain containing-3 (TIM-3), lymphocyte-activation gene 3 (LAG-3)), reduced production of effector cytokines (IFN-γ, tumor necrosis factor alpha (TNF-α), interleukin-2 (IL-2)), and diminished proliferative capacity. The T-cell receptor (TCR) of exhausted T cells interacts with tumor antigens presented on major histocompatibility complex class I (MHC-I), while PD-1 simultaneously engages with PD-L1, leading to inhibitory signaling. (**C**) **Regulatory T cell (T-reg) expansion:** The PD-1/PD-L1 interaction promotes the expansion and activation of T-regs in the TME. T-regs suppress effector T-cell responses through various mechanisms, including the production of immunosuppressive cytokines (IL-10, transforming growth factor beta (TGF-β), IL-35), direct cell-to-cell contact inhibition, and metabolic disruption (cluster of differentiation 39 (CD39/CD73)-mediated adenosine production). T-regs also interact with dendritic cells (DCs), inhibiting their ability to activate effector T cells. (**D**) **Suppression of innate immune cells:** PD-1/PD-L1 interactions affect innate immune cells in the TME. Tumor-associated macrophages (TAMs) expressing PD-1 are polarized towards an M2 phenotype upon interaction with PD-L1, leading to reduced pro-inflammatory cytokine production (TNF-α, IL-12) and increased immunosuppressive factors secretion (IL-10, TGF-β). Natural killer (NK) cells expressing PD-1 exhibit reduced cytotoxicity and IFN-γ production when engaging with PD-L1-expressing tumor cells. Created in BioRender. Kciuk, M. (2025) https://BioRender.com/bb5n1sm (accessed on 20 June 2025).

The immune checkpoint signaling involves recruitment of src homology 2 domain-containing phosphatase 2 (SHP2) upon PD-1 phosphorylation, leading to dephosphorylation of TCR-associated molecules, inhibition of cytokine production, and T-cell exhaustion (Figure 2).

## 3. PD-1/PD-L1 Inhibitors Approved by the FDA

### 3.1. Nivolumab

Nivolumab (MDX-1106 or BMS-936558), the first PD-1 inhibitor approved for colorectal cancer (CRC), was initially evaluated as a monotherapy in CRC patients in a study reported by Brahmer et al. in 2010. Among fourteen CRC patients, one complete response (CR) was observed [44]. In other phase 1 studies, the safety, anti-tumor activity, and pharmacokinetics of nivolumab were evaluated. However, there were no discernible objective responses observed [45,46]. The next clinical trial reported by Lipson et al. provided evidence for the effectiveness of nivolumab in one patient with CRC. A durable (3-year, ongoing) CR was observed following treatment [47]. In 2016, Hecht et al. reported that there was an upregulation of PD-L1 expression in patients with rectal adenocarcinoma following neoadjuvant radiochemotherapy. The observed increase implied that the combination of radiation and PD-1/PD-L1 pathway blockage could potentially serve as a promising therapeutic strategy for individuals in this patient population. Furthermore, the investigation revealed that there was a positive correlation between high levels of PD-L1 expression and a favorable outcome in randomized controlled trials (RCTs) including patients with rectal adenocarcinoma [48].

Squamous cell carcinoma of the anal canal (SCCA) is an atypical malignancy that is associated with human papillomavirus (HPV) and exhibits reduced responsiveness to conventional chemotherapy. Intratumoral HPV oncoproteins have been observed to increase the expression of PD-1 as a means to circumvent immune-mediated cytotoxicity. In the phase II clinical trial, nivolumab was well tolerated in patients. The study involved a total of 39 participants, out of which 37 were successfully recruited and administered at least one dosage of nivolumab. Of the 37 patients evaluated, 9 (approximately 24%) demonstrated an objective response, including 2 CRs and 7 partial responses (PRs). The adverse effects included anemia, fatigue, rash, and hypothyroidism. There were no instances of significant adverse effects reported [49]. Additionally, in a later case report, Cabel et al. provided evidence for the usefulness of HPV circulating tumor DNA as a noninvasive and early biomarker used to monitor the effectiveness of nivolumab and other immunotherapy treatments [50].

A phase 2 study revealed nivolumab’s promising potential in managing mCRC in patients with dMMR or MSI-H, both of which make tumors more likely to respond to immunotherapy. The study showed that nivolumab provided lasting disease control in mCRC patients who had already undergone conventional treatments with fluoropyrimidine, oxaliplatin, or irinotecan. This represents a major advancement, as PD-1 inhibitors like nivolumab give dMMR/MSI-H mCRC patients an effective alternative when standard chemotherapy regimens fall short [51].

A better understanding of the MMR mechanism is essential in order to grasp why nivolumab works in this subset of mCRC. MMR is a DNA repair pathway critical to the recognition and correction of replication errors. When MMR function is compromised—either due to mutations in the coding of the genes for MMR proteins or due to hypermethylation of MMR gene promoters—the system’s ability to detect and fix replication errors is significantly reduced [52]. A deficiency in the MMR system allows genetic mutations to build up, especially in repetitive DNA sequences known as microsatellites. This accumulation, known as MSI-H, involves frequent mutations in these regions, often more than 12 per million DNA bases [53,54,55,56,57]. Genetic instability observed in dMMR/MSI-H cancers plays a role in their responsiveness to immune checkpoint inhibitors (ICIs). This instability is due to the accumulation of mutations in the tumor’s DNA, which lead to abnormal proteins being expressed on the surface of the tumor cells, marking them as foreign to the immune system. Two main methods, immunohistochemistry (IHC) and polymerase chain reaction (PCR), are used to detect the dMMR/MSI-H and determine which patients are most likely to benefit from immunotherapy. IHC measures the presence of MMR proteins such as MutL homolog 1 (MLH1), MutS homolog 2 (MSH2), MutS homolog 6 (MSH6), and postmeiotic segregation increased 2 (PMS2), with a deficiency indicating dMMR. PCR, on the other hand, assesses the length of microsatellite sequences in tumor tissue relative to normal tissue, where significant discrepancies confirm MSI-H [58]. Biological factors such as gender can affect the effectiveness of PD-1 antibodies in CRC and other types of cancer. Findings have shown that male patients respond better to PD-1 antibodies, compared to female patients, as observed in cancers such as melanoma and non-small cell lung cancer [59]. Women generally exhibit more robust immune responses compared to men, which may contribute to the higher incidence of autoimmune diseases in females but could also influence the efficacy and tolerability of ICIs in cancer therapy [60].

The findings of one of the first clinical trials indicated that nivolumab monotherapy was well tolerated and resulted in sustained responses and disease control. All individuals who responded to the treatment were still alive at the time of data analysis, suggesting long-term survival in a group of dMMR/MSI-H mCRC patients with pre-existing treatments. The study examined the effects of the drug on dMMR/MSI-H mCRC patients belonging to various subgroups, including those with positive (≥1%) or negative (<1%) tumor PD-L1 expression, tumors containing serine/threonine-protein kinase B-Raf (*BRAF*) or Kirsten rat sarcoma viral oncogene homolog (*KRAS*) mutations, and patients with or without a clinical history of Lynch syndrome. The study showed positive responses and disease control in these patients. Furthermore, nivolumab exhibited significant enhancements in QoL among individuals diagnosed with dMMR/MSI-H mCRC [51].

Based on observations that the efficacy of specific ICIs is limited by the up-regulation of other immune checkpoint molecules (for instance, the up-regulation of PD-1 following the administration of anti-cytotoxic T-cell antigen 4 (CTLA-4) antibodies), combination strategies involving ICIs were indicated as a feasible approach for enhancing the therapeutic response. The preclinical investigations demonstrated that the co-administration of ipilimumab (CTLA-4 blocker) and nivolumab resulted in augmented anticancer effects in two syngeneic tumor mouse models. The investigation employed immunohistochemistry, flow cytometry, and cytokine analysis techniques to examine alterations in the TME following antibody therapy. The findings of the study indicated that the administration of combination therapy resulted in a significant augmentation of CD8+ T-cell infiltration and a concomitant reduction in the population of T-reg cells in the TME. Moreover, the utilization of combination therapy resulted in a notable increase in the synthesis of INF-γ and other cytokines, which exhibited a correlation with immune responses against tumor cells. The results of this study offered a preclinical justification for employing ipilimumab and nivolumab in combination therapy [61].

The combination of nivolumab and ipilimumab exhibited notable response rates, promising PFS and OS following treatment, a tolerable safety profile, and significant enhancements in important patient-reported outcomes. Based on the study, it was concluded that the utilization of combination therapy exhibits enhanced effectiveness compared to the use of anti-PD-1 monotherapy. Furthermore, combination therapy demonstrated a good benefit–risk profile and has been identified as a potentially effective therapeutic approach for individuals diagnosed with dMMR/MSI-H mCRC [62,63,64,65,66,67].

Similarly, the coadministration of regorafenib at a dosage of 80 mg in conjunction with nivolumab demonstrated a tolerable safety profile, while also exhibiting promising anticancer activity in individuals diagnosed with gastric and CRC cancers [68]. Nevertheless, a phase I/IIb study including individuals diagnosed with MSS CRC showed that only four (10%) of 40 evaluated patients had PRs following combination treatment of regorafenib and nivolumab, while 21 (53%) had stable disease (SD), and the DCR was 63%. The median PFS was 4.3 months and the median OS 11.1 months [69]. Another study, comprising 84 patients receiving a combination of regorafenib and a PD-1 antibody (nivolumab (35 patients), toripalimab (35 patients), sintilimab (11 patients), or camrelizumab (3 patients)), and 95 receiving regorafenib alone showed that administering regorafenib in combination with PD-1 antibodies led to a significantly longer PFS, compared to using regorafenib alone. This was observed in both male and female patients who did not have liver metastases. Female patients with liver metastases who received a combination of regorafenib and a PD-1 antibody had a shorter PFS compared to those who received regorafenib alone. Liver metastasis is a significant clinical challenge in CRC, often impairing PD-1 antibody efficacy. Metastases in the liver have been shown to create an immunosuppressive environment by activating regulatory T cells (T-regs) and depleting CD8+ T cells, both of which are critical to effective anti-tumor immunity. As a negative prognostic factor, liver metastasis necessitates additional strategies to enhance immune response in affected patients, potentially through combination therapies [59].

Among American patients (n = 70) with MSS/proficient MMR (pMMR) mCRC, the combination of regorafenib and nivolumab resulted in an ORR of 7%. This response rate was observed in patients without liver metastases. The investigation revealed a slightly higher occurrence of grade 3 rash; however, apart from that, the safety characteristics of the combination of regorafenib and nivolumab were manageable and consistent with the published safety profiles of both drugs [70].

CRC that is characterized by MSS exhibits a very low immunogenicity, hence presenting a challenge in terms of available therapeutic interventions. Radiotherapy and chemotherapy treatments are known to cause DNA damage in cancer cells, ultimately resulting in their eradication. However, recent studies have yielded evidence that DNA damage response (DDR) has a substantial role in influencing the efficacy of cancer immunotherapy, as discussed in our earlier work [70]. This can be attributed to the elevated expression of PD-L1 on cancer cells following chemotherapy/radiotherapy use, prompting an increased production of epitopes on cancer cells, to which anti-PD-L1 antibodies can bind [71].

Trifluridine (TFD) is a nucleoside analog derived from thymidine that exerts its inhibitory effects on cell proliferation by integrating into DNA, and hence inducing malfunction in the DNA structure [72]. In contrast, tipiracil (TPI) functions as a potent inhibitor of thymidine phosphorylase, hence facilitating the preservation of trifluridine levels in the bloodstream. Inhibition of the thymidine phosphorylase enzyme effectively prevents the deactivation of trifluridine [73]. The combination of TFD/TPI and PD-1 inhibitors in MSS CRC xenograft models showed a synergistic effect in terms of anti-tumor efficacy and enhanced tumor immunogenicity. Nevertheless, the clinical outcomes of patients with refractory MSS mCRC were not improved by the combination of TFD/TPI and nivolumab. The results from this study demonstrated that the combination treatment was well-tolerated and feasible for this particular patient population [74]. Similarly, promising outcomes were achieved when the combination was supplemented with another DNA-damaging agent, oxaliplatin. Oxaliplatin’s mechanism of action involves the formation of covalent bonds with DNA, leading to the formation of DNA crosslinks. These crosslinks inhibit DNA replication and transcription in rapidly dividing cells. The combination of TFD/TPI along with oxaliplatin and either nivolumab or bevacizumab exhibited a satisfactory safety profile and anti-tumor activity in patients with mCRC who had received prior treatment [75].

A non-randomized, single-arm phase II trial was conducted to evaluate the efficacy of a treatment regimen comprising radiation, ipilimumab, and nivolumab, in patients diagnosed with MSS mCRC. The incorporation of radiation therapy alongside the inhibition of the PD-1 and cytotoxic T-lymphocyte-associated protein 4 (CTLA4) pathways exhibited certain efficacy in patients with resistant CRC, who have historically shown limited response to simultaneous PD-1 and CTLA4 pathway inhibition [76].

The results of the 2022 MAYA clinical trial indicated that temozolomide (TMZ) may enhance the sensitivity of patients with methylguanine methyltransferase (*MGMT*)-silenced, MSS CRC to the combination of ipilimumab and nivolumab [77]. MGMT is a DNA repair protein that counteracts the cytotoxic effects of alkylating agents, such as TMZ, by removing alkyl groups from the O6 position of guanine, thereby restoring DNA integrity. Interestingly, the ability of TMZ to sensitize tumors to ICIs appears to be independent of *MGMT* status. This observation suggests that the therapeutic potential of combining TMZ with ICIs may extend to a broader patient population, including MSS CRC patients with tumors that express MGMT. Further work is required to understand the mechanism by which short-term TMZ treatment enhances sensitivity to ICIs. Possible explanations may involve the stimulation of cancer cells or immune cells to secrete immunostimulatory cytokines as a result of TMZ treatment, or the modulation of immunomodulatory receptors or ligands such as PD-1/PD-L1 due to TMZ treatment [78].

CD27, belonging to the TNF receptor superfamily, has a crucial function in T-cell activation by delivering an additional co-stimulatory signal. Stimulating CD27 using anti-CD27 mAbs enhances the immune response of cytotoxic T cells (CTLs) against tumors. Prior research has shown that anti-CD27 agonistic mAbs can effectively impede the growth of tumors in various mouse models of both solid and hematological malignancies [79,80]. Sanborn et al. performed a phase 1/2 dose-escalation and expansion study of a varlilumab (anti-CD27 mAb) and nivolumab combination in advanced solid tumors, including CRC. The trial revealed that the concurrent administration of an agonist anti-CD27 antibody (varlilumab) and nivolumab was well-tolerated in patients with advanced solid malignancies. The combination therapy exhibited a satisfactory safety profile, with side effects that were easily controlled. However, no increase in tumor PD-L1 expression and T-cell infiltrates (CD8+, CD4+, and FOXP3+ cells) was observed in the on-study biopsy of patients derived from the CRC cohort [81].

Similarly, the glucocorticoid-induced tumor necrosis factor receptor-related protein (GITR) is expressed on the surface of T cells, with its expression upregulated upon cellular activation. GITR functions as a co-stimulatory receptor that enhances T-cell activation and proliferation, and it has been implicated as a potential target in cancer immunotherapy [82]. Studies have demonstrated that GITR signaling enhances both cellular and humoral immune responses. In murine cancer models, GITR activation has been shown to mitigate immunosuppressive activity mediated by regulatory T cells (Tregs), thereby promoting anti-tumor immunity [83]. Preclinical evidence indicates that the use of GITR agonists, along with T-reg depletion, can improve the ratio of intratumoral effector T cells and T-reg cells, leading to the reduction of tumor size [84]. The study by Rakké et al. showed that GITR is mostly found on tumor-infiltrating lymphocytes (TILs) in patients with pMMR CRC and mCRC. Specifically, it is found on CD8+ TILs that are functionally impaired and have increased expression of inhibitory receptors and TOX (a transcription factor that has been associated with T-cell exhaustion and has been implicated in regulating the function of exhausted T cells within the TME). Furthermore, the expression of GITR is increased in TILs relative to peripheral blood mononuclear cells (PBMCs) and nearby tissues. This expression is mainly detected in activated T-reg cells within the CD4+ TIL population and potential tumor-reactive CD103+ CD39+ TILs among the CD8+ TIL population. These findings suggest that GITR plays a regulatory role in modulating immune responses within the TME. Furthermore, the study demonstrates that activation of the GITR pathway enhances the functionality of TILs and synergizes with anti-PD-1 therapy to promote their reinvigoration. This leads to increased TIL proliferation and elevated secretion of pro-inflammatory cytokines and chemokines. Collectively, the results indicate that targeting GITR may potentiate anti-tumor immune responses in patients with pMMR CRC and colorectal liver metastases [85].

### 3.2. Pembrolizumab

Phase 2 research was conducted to assess the clinical effectiveness of pembrolizumab in 41 patients with progressing metastatic cancer, with or without dMMR. Pembrolizumab was delivered intravenously at a dosage of 10 mg per kilogram of body weight every 14 days. The main objectives of the study were to determine the immune-related ORR and the 20-week immune-related PFS rate. The ORR and PFS related to the immune system were 40% and 78%, respectively, for CRCs with a deficiency in MMR, and 0% and 11% for CRCs with pMMR [54]. Subsequent studies in the cohorts of patients with PD-L1-positive advanced SCCA [86] and advanced CRC [87] showed acceptable safety and the promising anti-tumor efficacy of pembrolizumab, warranting further investigations. The KEYNOTE-164 (NCT02460198) study assessed the effectiveness of pembrolizumab in treating MSI-H/dMMR mCRC. This phase II investigation was conducted in 128 locations across the globe. Patients received at least two previous lines of standard therapy, which included fluoropyrimidine, oxaliplatin, and irinotecan, with or without anti-VEGF/EGFR mAb (cohort A). Alternatively, patients in cohort B were administered pembrolizumab at a dosage of 200 mg every 3 weeks for a maximum duration of 2 years, or until the occurrence of disease progression, intolerable side effects, or voluntary discontinuation. The median follow-up period for cohort A was 31.3 months, while for cohort B it was 24.2 months at the data cutoff. The ORR was 33% in both cohorts, and the duration of response (DoR) was not determined in either cohort. The median PFS values were 2.3 months and 4.1 months, respectively. The median OS was 31.4 months, while the maximum survival time has not been determined. The trial determined that pembrolizumab is efficacious and demonstrates a controllable safety profile in patients diagnosed with MSI-H/dMMR CRC [88]. In a subsequent study, pembrolizumab was demonstrated to significantly prolong PFS compared to chemotherapy when used as the initial treatment for mCRC with MSI-H and dMMR. Additionally, pembrolizumab resulted in fewer side events linked to treatment [89]. These findings, combined with the later reported clinical benefits in QoL and symptom management compared to chemotherapy, supported the use of pembrolizumab as a preferred first-line treatment option for patients with MSI-H or dMMR mCRC, underscoring the advantages of immunotherapy over conventional chemotherapy [90]. As a result, pembrolizumab was granted FDA approval on 29 June 2020, for the treatment of patients diagnosed with unresectable or MSI-H mCRC who have not had any previous systemic treatment employed. The permission was granted based on the data obtained from Study Keynote-177 [91], which ended in 2022 [92].

Pembrolizumab was also investigated in trials involving various combinations with other therapeutic approaches. These include the use of chemotherapy including 5FU/leucovorin/oxaliplatin [93,94], TMZ [95], EGFR inhibitors such as cetuximab [96], DNA methyltransferase inhibitor (azacitidine) [97], antiangiogenic agents (ziv-aflibercept) [98], C-C motif chemokine receptor 5 (CCR5) inhibitor (maraviroc) [99], and C-X-C motif chemokine receptor 2 (CXCR2) inhibitor (navarixin) [100].

In a recent study, the combination of allogeneic whole-cell granulocyte–macrophage colony-stimulating-factor-secreting immunotherapy (Colon GVAX) with a low dose of alkylating agent, cyclophosphamide, and pembrolizumab was evaluated in patients with advanced pMMR CRC. This single-arm trial enrolled 17 patients and assessed the ORR, OS, PFS, and immune-related correlates. Despite these efforts, no objective responses were observed, resulting in a DCR of 18%. The median PFS was 82 days, while the median OS reached 213 days. Although these results indicate that the trial’s primary objective was not met, an intriguing finding emerged with biochemical responses (≥30% decline in carcinoembryonic antigen (CEA)) in 41% of patients. This phenomenon, coupled with observed increases in PD-L1 expression and tumor necrosis in a subset of patients, suggests a potential immune modulation effect of GVAX/cyclophosphamide. However, significant treatment-related adverse events (grade ≥ 3) such as hemolytic anemia and corneal transplant rejection were reported in two patients. Ultimately, while this trial demonstrated that the addition of GVAX/cyclophosphamide to pembrolizumab did not achieve significant clinical responses in pMMR CRC, the observed biochemical changes highlight an area for further exploration into combined immunotherapy strategies [101].

In a phase I/II trial assessing the combination of napabucasin (signal transducer and activator of transcription 3 (STAT3) pathway inhibitor) and pembrolizumab for mCRC, researchers explored efficacy and safety outcomes. The trial commenced with phase I, which aimed to determine the recommended phase II dosage (RP2D) for napabucasin, using a dose-escalation approach. The doses ranged from 240 to 480 mg, taken twice daily, alongside the administration of 200 mg of pembrolizumab every three weeks. The phase II segment included two cohorts: cohort A (10 patients with MSI-H CRC) and cohort B (40 patients with MSS CRC). The primary endpoint was the immune-related objective response rate (irORR), with additional analyses involving PD-L1 expression, combined positive score (CPS), genomic profiles, and the consensus molecular subtypes (CMS) of CRC. The trial enrolled a total of 55 patients, and determined that napabucasin 480 mg was the optimal RP2D after phase I, as no dose-limiting toxicities were observed. In phase II, the irORR was 50% for cohort A and 10% for cohort B. Within cohort B, response rates varied with PD-L1 CPS, with irORRs of 0%, 5.3%, and 42.9% in CPS < 1, 1 ≤ CPS < 10, and CPS ≥ 10, respectively. Furthermore, a higher TMB correlated with better response rates, suggesting a potential biomarker for predicting outcomes. The investigation introduced CMS classification in 18 evaluable patients from cohort B, revealing irORRs of 33.3% in CMS1, 0% in CMS2, 33.3% in CMS3, and 33.3% in CMS4. While the trial did not achieve its primary endpoint, it did show anti-tumor activity, along with an acceptable safety profile, suggesting that the combination of napabucasin and pembrolizumab has potential for both MSS and MSI-H mCRC, with further investigation needed to explore biomarkers and the specific mechanisms driving response rates [102].

A phase I clinical trial evaluated MK-1248, a humanized anti-GITR mAb agonist, as monotherapy and in combination with pembrolizumab, in patients with advanced solid tumors. The trial, conducted using a 3 + 3 dose-escalation design, aimed to determine the safety and tolerability, maximum tolerated dose (MTD), and pharmacokinetics/pharmacodynamics of the treatment. MK-1248 was administered intravenously every three weeks, with the monotherapy treatment given for up to four cycles, and the combination therapy with pembrolizumab for up to 35 cycles. A total of 20 patients received MK-1248 as monotherapy, and 17 received it in combination with pembrolizumab. The most common tumor types were CRC, melanoma, and renal cell carcinoma. MK-1248 was well tolerated at the highest doses of 170 mg monotherapy and 60 mg combination treatment. The study reported no dose-limiting toxicities or treatment-related deaths, indicating an overall favorable safety profile. Vomiting, anemia, and decreased appetite were the most common side effects in 36 of 37 participants. Grade 3 to 5 adverse effects occurred in 51% of patients, with treatment-related adverse effects reported in 49% of the cohort. The trial found that the combination therapy with pembrolizumab had some anti-tumor activity, with one patient achieving a CR and two achieving a PR, resulting in an ORR of 18%. Monotherapy with MK-1248, however, did not yield any objective responses. The DCR (SD or better) was 15% for monotherapy and 41% for combination therapy. In conclusion, MK-1248 showed a tolerable safety profile, with no dose-limiting toxicities or treatment-related deaths at the tested doses. However, the combination therapy with pembrolizumab demonstrated limited anti-tumor activity, suggesting that while the safety profile is acceptable, the efficacy of the combination may require further investigation and optimization to improve patient outcomes in advanced solid tumors [103].

### 3.3. Dostarlimab (TSR-042)

The first reports on the clinical efficacy of dostarlimab appeared in 2022. Twelve patients had successfully undergone treatment with the antibody and had been monitored for a minimum of 6 months. All 12 patients exhibited a clinical CR, indicating the absence of any tumor evidence. None of the patients had undergone chemoradiotherapy or surgery, and there were no instances of progression or recurrence recorded during the follow-up periods, which ranged from 6 to 25 months. There have been no recorded occurrences of severe adverse effects, namely, those classified as grade 3 or higher [104]. In later reports of dostarlimab (phase II study), all 14 monitored patients with locally advanced rectal cancer that had a dMMR exhibited a clinical full response, without requiring any additional therapy. While the initial clinical efficacy of dostarlimab in patients with locally advanced rectal cancer exhibiting a dMMR phenotype is promising, several limitations exist. The absence of a control group in the study raised concerns about the reliability of the results, as it limited the ability to compare outcomes against traditional treatments, such as chemoradiotherapy and surgery. This lack of a control group also created a risk of bias, given the remarkable success of the treatment, leading to “off-protocol” use of neoadjuvant immunotherapy and potentially reducing the number of patients willing to enroll in future randomized studies. Additionally, the follow-up period and the DoR in the reported studies are relatively short. Although no severe adverse events (grade 3 or higher) were observed, and none of the patients required additional therapy, half of the patients were monitored for less than a year. This limited follow-up makes it difficult to assess the long-term efficacy and safety of dostarlimab. Critical endpoints such as 3-year OS were not reported, leaving significant gaps in the understanding of the treatment’s potential impact. Moreover, all patients in the initial reports were enrolled at a single institution, Memorial Sloan Kettering Cancer Center, known for its extensive expertise in the nonoperative management of rectal cancer. This single-center approach may introduce institutional biases and limit the generalizability of the results. The analysis of data from multiple institutions is essential to validate these findings and ensure they apply across diverse clinical settings. Despite these limitations, the study has garnered praise for its innovative approach and potential impact on the treatment of dMMR CRC. Dr. Ng, who commented on the study, acknowledged that while the idea of using immunotherapy in a potentially curative setting “*may seem bold at first*,” the research is backed by robust data demonstrating the high efficacy of checkpoint blockade in treating MMR-deficient mCRC [105].

The GARNET was a phase 1 clinical study that started enrolling participants on 8 May 2017. It was an open-label trial conducted at multiple research centers. In the study, a total of 327 individuals diagnosed with advanced or recurrent dMMR and MSI-H or polymerase epsilon (*POLE*)-mutated solid tumors were enrolled. Among them, 32.1% had dMMR CRC. The data used for this interim study were collected from 1 November 2021, and the median follow-up period was 27.7 months. Patients were administered 500 mg of dostarlimab intravenously at three-week intervals for a total of four doses. Afterward, the dosage was increased to 1000 mg every six weeks until disease progression, cessation, or withdrawal. Dostarlimab demonstrated excellent tolerability and exhibited quick, strong, and long-lasting anticancer effects in patients with various dMMR solid tumors, which led to its approval in 2023 [106]. Dostarlimab is currently being investigated in clinical trials in various combinations involving cobolimab (TSR-022; TIM-3 inhibitor) (NCT02817633), niraparib (poly (ADP-ribose) polymerase inhibitor; PARPi) (NCT06365970), and hypofractionated radiotherapy (NCT04926324), or GSK4381562 (anti-PVRIG; PVR Related Immunoglobulin Domain Containing Protein; CD112R antibody) and GSK4428859A (anti-T-cell immunoglobulin and immunoreceptor tyrosine-based inhibitory motif domain (TIGIT) antibody) (NCT05277051) treatments.

## 4. Inhibitors Currently Not Approved for CRC Treatment

### 4.1. AMP-224

AMP-224 is a recombinant fragment crystallizable (Fc) fusion protein that binds to the PD-1 receptor. It was evaluated in combination with low-dose cyclophosphamide and stereotactic body radiation therapy (SBRT) in patients with mCRC who were refractory to standard chemotherapy. A total of fifteen patients were enrolled. Six patients received SBRT at a dose of 8 Gy on day 0 (designated as dose level 1), while nine patients received 8 Gy on days −2 to 0 (designated as dose level 2). Cyclophosphamide was administered intravenously at a dose of 200 mg/m^2^ on day 0. Additionally, all patients received AMP-224 at a dose of 10 mg/kg intravenously on day 1, with subsequent doses given biweekly for a total of six infusions. The primary objectives of the study were to evaluate the feasibility and safety of this combinatorial therapeutic approach. The main objectives of the study were to assess the feasibility and safety of the intervention. Ten individuals (67%) successfully finished six doses of AMP-224, while five patients (33%) had to terminate treatment because their condition had worsened. No instances of dose-limiting toxicity were detected. Out of the total number of patients, 9 individuals (60%) encountered treatment-related adverse events, all of which were classified as Grade 1 or 2. The median PFS and OS were 2.8 months and 6.0 months, respectively. The pre-treatment tumor biopsy samples exhibited M2 macrophage polarization, whereas the post-treatment samples did not. The combination of AMP-224, SBRT, and low-dose cyclophosphamide was well tolerated but did not result in any substantial clinical improvement in patients with mCRC [107].

### 4.2. Atezolizumab

The clinical efficiency and safety of atezolizumab were reviewed by Rico and Price in 2018. Therefore, we have focused on later studies published after the release of this excellent work [108]. Preclinical studies have shown that inhibition of mitogen-activated protein kinase kinase (MEK) can promote the expansion and persistence of tumor-specific T lymphocytes within the TME, and may act synergistically with ICIs to enhance anti-tumor immune responses [109]. IMblaze 370, a multicenter, open-label, phase 3, randomized, controlled trial was conducted to evaluate the combination of atezolizumab plus cobimetinib (MEK inhibitor) in 365 mCRC patients (183 patients in the atezolizumab and cobimetinib group, 90 in the atezolizumab group, and 90 in the regorafenib group). The IMblaze370 trial did not meet its primary endpoint of improved overall survival (OS) when comparing atezolizumab in combination with cobimetinib or, as monotherapy, to regorafenib, in patients with metastatic colorectal cancer. The safety profile of the atezolizumab and cobimetinib combination was comparable to that observed with each agent being administered individually [110,111]. Germani and Moretto provided a critical review of two atezolizumab clinical trials. The AtezoTRIBE [112] trial compared the effects of adding atezolizumab to FOLFOXIRI (a combination of 5FU, oxaliplatin, and irinotecan) and bevacizumab in a group of previously untreated mCRC patients. This study builds upon the concept of the immunogenic cell death (ICD) induced by chemotherapy, which leads to the release of tumor-associated neoantigens. These neoantigens are subsequently captured by DCs, which process and present them to CTLs, thereby initiating an adaptive immune response against tumor cells. Bevacizumab inhibits the VEGF/VEGFR pathway, leading to vascular normalization, which in turn promotes increased infiltration of CTLs into the tumor. Furthermore, anti-angiogenic drugs promote the development of DCs and inhibit the proliferation of T-regs and myeloid-derived suppressor cells (MDSCs). This ultimately leads to the activation of immunological effector cells. In general, these pathways can alter the immunological milieu in a way that promotes an immune response and consequently enhances the effectiveness of ICIs. The study found that this combination provided a substantial advantage in terms of PFS, regardless of the patients’ MMR/MSI status. The single-arm MAYA trial [77] demonstrated that immune priming with TMZ in pMMR/MSS chemo-resistant mCRC patients who had silenced *MGMT* resulted in indications of responsiveness to the subsequent treatment with nivolumab plus a low dosage of ipilimumab, in certain patients. Enhanced sensitivity to TMZ can be observed when the promoter of *MGMT* is inactivated through hypermethylation. TMZ sensitivity was primarily shown in tumors with dMMR or MSS CRC and total loss of MGMT protein. Following the initial response to therapy, resistance to TMZ may arise due to re-expression of *MGMT* or the selective expansion of *MGMT*-expressing subclones. Additionally, acquired mutations in MMR genes can lead to a hypermutated phenotype, potentially increasing the sensitivity of mCRC to ICIs [113]. In a double-blind phase 2 RCT, the addition of atezolizumab to a treatment regimen of capecitabine and bevacizumab resulted in a modest improvement in PFS (median, 4.4 months vs. 3.6 months with placebo). Although this difference met the predefined threshold for statistical significance, it was not deemed clinically meaningful [114]. Similarly, the addition of atezolizumab to bevacizumab as a first-line maintenance treatment following FOLFOX + bevacizumab introduction for *BRAFwt* mCRC did not result in any improvement in effectiveness outcomes [115].

There are currently 23 phase I, 37 phase II, and 9 phase III clinical trials registered on https://clinicaltrials.gov/ (accessed on 7 May 2025) that investigate atezolizumab for CRC treatment (Table 1).

### 4.3. Avelumab

Avelumab has been shown to activate both adaptive and innate immune responses. Unlike other mAbs targeting the PD-1/PD-L1 axis, avelumab retains an intact Fc region, enabling antibody-dependent cell-mediated cytotoxicity (ADCC). Among ICIs, it had the shortest clinical development timeline, achieving regulatory approval within 52 months of IND submission [116]. The clinical trial of avelumab in patients with mCRC showed no observable positive outcomes, and the median duration of time without disease progression was 2.1 months. In addition, the study found that the majority of patients (53%) had SD, while the remaining patients (47%) had “progressing disease” as their best overall response. These findings indicated that avelumab does not demonstrate substantial anticancer efficacy in a group of patients with mCRC. Moreover, the safety characteristics of avelumab were in line with those of other mAbs targeting PD-1/PD-L1, as evidenced by several patients encountering grade 3 treatment-related side effects such as hepatoxicity, lymphopenia, and amylase/lipase elevation. Ultimately, the study determined that avelumab did not demonstrate efficacy in generating objective responses in a broad range of patients with mCRC. This underscored the necessity for additional investigation and the creation of treatment combinations to enhance the immune system response in this particular group of patients [117].

In a phase I/II multicenter study (GEMCAD 1602), the concurrent administration of avelumab and autologous dendritic cell (ADC) vaccination in pre-treated mCRC patients who had MSS tumors was shown to be safe and well-tolerated; however, it demonstrated only limited clinical efficacy [118].

In contrast, a recent RCT examined the effectiveness and safety of avelumab as a second-line treatment for patients with mCRC characterized by dMMR/MSI status. These patients had not received immunotherapeutic agents beforehand and had failed standard first-line treatment. The trial found that avelumab treatment resulted in significantly improved PFS and DCR, compared to standard second-line treatment. Furthermore, avelumab had a positive safety profile when used as a second-line treatment for dMMR/MSI mCRC [119]. Table 2 summarizes the findings of clinical trials of avelumab for CRC treatment (Table 2).

There are currently seven phase I and twenty-one phase II clinical trials, and one terminated phase III clinical trial, registered on https://clinicaltrials.gov/ (accessed on 7 May 2025) investigating avelumab for CRC treatment.

### 4.4. Camrelizumab

The combined administration of preoperative short-course radiotherapy, chemotherapy (capecitabine and oxaliplatin), and camrelizumab demonstrated both efficacy and safety in patients with locally advanced rectal cancer. The study reported a pathological complete response (pCR) in 48.1% of patients. The regimen was well tolerated, with no grade 4 or 5 adverse events observed [120].

A retrospective study examining the effectiveness and safety of camrelizumab used in combination with XELOX (capecitabine and oxaliplatin), in combination with bevacizumab or regorafenib, for patients with mCRC, showed significant efficacy. Specifically, 72% of patients achieved a PR and 24% achieved SD. The ORR was 72% while the DCR was 96%. The median PFS was 11.2 months, whereas the OS has not yet been determined. The study showed that the combination of camrelizumab with XELOX plus bevacizumab or regorafenib both was feasible and resulted in a high rate of responses when used as the treatment in MSS mCRC Chinese patients, regardless of patient selection. The authors emphasized the need for prospective RCTs with larger sample sizes to further validate these findings [121].

A recent clinical trial assessed the effectiveness and safety of combining cetuximab with camrelizumab and liposomal irinotecan in patients with RAS wt mCRC who had previously received anti-EGFR-based treatment. Patients diagnosed with RASwt mCRC who had undergone a minimum of two previous systemic treatments, including anti-EGFR-based therapy for metastatic or unresectable CRC, were included in the cohort. The patients received intravenous treatment once every 2 weeks, consisting of cetuximab at a dose of 500 mg/m^2^, camrelizumab at a dose of 200 mg/m^2^, and liposomal irinotecan (HR070803) at a dose of 60 mg/m^2^. The ORR was 25% and the DCR was 75%. The median PFS and OS were 6.9 and 15.1 months, respectively. 15.8% of patients experienced grade 3 treatment-related adverse events. Cetuximab in combination with camrelizumab and HR070803 as a retreatment therapy for patients with RASwt mCRC showed promising results as a late-line treatment option. This treatment has demonstrated effective anti-tumor activity and was well-tolerated, with manageable toxicity [122] (Table 3).

A phase III clinical trial (NCT06229041) is currently underway to evaluate the efficacy and safety of camrelizumab in combination with total neoadjuvant treatment (TNT) in patients with high-risk locally advanced rectal cancer (LARC). The primary objective is to compare the pCR rate between patients receiving TNT alone and those receiving TNT combined with camrelizumab. Secondary endpoints include three-year disease-free survival (DFS) and the assessment of chemoradiotherapy and immunotherapy-related toxicities. The trial, which began on 29 March 2023, plans to enroll 472 participants and is estimated to be completed by 1 October 2029. Postoperative complications and outcomes will also be evaluated, providing comprehensive data on the potential benefits of adding immunotherapy to standard neoadjuvant treatment regimens.

Similarly, the Union trial (NCT04928807) is a phase III study designed to evaluate the efficacy and safety of neoadjuvant short-course preoperative radiotherapy (SCPRT) combined with camrelizumab and chemotherapy in patients with locally advanced rectal cancer (LARC). This trial aims to compare the outcomes of SCPRT with camrelizumab and chemotherapy, and the standard long-course chemoradiotherapy (LCCRT) followed by chemotherapy. Eligible patients with clinical stage T3-4/N+ rectal adenocarcinoma are randomized in a 1:1 ratio to either arm A (SCPRT, camrelizumab, and chemotherapy) or arm B (LCCRT and chemotherapy). The primary endpoint is pCR, while secondary endpoints include 3-year event-free survival, OS, R0 resection rate, and QoL. Additionally, safety is assessed using the National Cancer Institute’s Common Terminology Criteria for Adverse Events. The trial began enrollment in July 2021, with a planned enrollment of 230 patients. This study will provide important data comparing the novel approach of the combination of immunotherapy with radiotherapy and chemotherapy to the established LCCRT regimen, potentially improving short- and long-term outcomes for patients with high-risk LARC.

### 4.5. Durvalumab

In 2015, Stewart et al. reported the first evidence of durvalumab (MEDI4736) effectiveness in the xenograft model of CRC. Anti-mouse PD-L1 demonstrated a substantial enhancement in the survival rate of mice grafted with CT26 CRC cells. The combination of anti-PD-L1 and oxaliplatin boosted the anticancer efficacy of the treatment by promoting the release of high mobility group box 1 (HMGB1), which acts as a pro-inflammatory factor [123]. In a phase II single-arm study, patients were treated with a combination of durvalumab (1500 mg), tremelimumab (75 mg), and radiation, administered every 4 weeks. The main focus of the study was to determine the ORR in lesions that were not treated with radiation. The study did not fulfill the predetermined criteria for the endpoint, indicating that it was not feasible for future investigation. Nevertheless, there were occasional occurrences of systemic immune enhancement and reduction in non-irradiated lesions (abscopal response), while at the same time, the combination exhibited safety [124]. Similarly, in a study evaluating the combination of neoadjuvant durvalumab and tremelimumab in the preoperative control of resectable CRC liver metastases, the combination demonstrated safety and showed enhanced anti-tumor immune response, as evidenced by the activation of B and T cells [125]. The administration of durvalumab at a dosage of 1500 mg every 4 weeks through intravenous infusion demonstrated favorable clinical effectiveness, with promising rates of response and satisfactory results in terms of survival in patients with mCRC who had MSI-H/dMMR or mutations in the POLE exonuclease domain (POLE EDM). The study found that patients with *POLE*-mutated mCRC may experience a clinical response to durvalumab [126]. A phase I/II study assessed the safety and effectiveness of combining pexastimogene devacirepvec (PexaVec) with durvalumab, both with and without tremelimumab, in patients with mCRC who were resistant to standard chemotherapy and had pMMR. The trial included a total of 34 patients with mCRC: 16 patients were assigned to the PexaVec/durvalumab cohorts, and 18 patients were assigned to the PexaVec/durvalumab/tremelimumab groups. In general, the administration of PexaVec in conjunction with ICIs did not yield unexpected adverse effects. The median PFS in the PexaVec/durvalumab/tremelimumab groups was 2.3 months compared to 2.1 months in the PexaVec/durvalumab groups [127]. The MEDITREME trial, a phase 1b/2 study, examined the safety and effectiveness of combining durvalumab and tremelimumab with 5-FU, leucovorin, and oxaliplatin (mFOLFOX6) chemotherapy as a first-line treatment in 57 patients with *RAS*-mutated unresectable mCRC. The phase Ib study showed the safety of the combination, while the 3-month PFS in patients with MSS tumors was successfully achieved in phase 2. The 3-month PFS rate was 90.7%. Responders had an elevated TMB and reduced genomic instability. An integrated transcriptome study revealed that a strong immunological signature and a low level of epithelial–mesenchymal transition were correlated with a more favorable result. The concurrent administration of durvalumab–tremelimumab with mFOLFOX6 demonstrated favorable tolerability and showed encouraging clinical efficacy in patients with MSS mCRC [128]. In the PANDORA trial the effectiveness and safety of preoperative treatment with durvalumab at a dose of 1500 mg every 4 weeks for three administrations after a course of radiotherapy (a total dose of 5040 cGy delivered in 25–28 fractions for 5 weeks), which was accompanied by the simultaneous use of capecitabine (at a dose of 825 mg/m^2^ twice daily), was assessed. The main objective of the study was to determine the rate of pCR. Secondary objectives included assessing the proportion of clinical full remissions and evaluating the treatment safety. Two individuals exhibited illness progression during treatment. Out of the 55 eligible patients, 19 of them achieved a pCR (34.5% of patients). The toxicity associated with durvalumab use included grade 3 adverse events (diarrhea, skin toxicity, transaminase elevation, lipase increase, and pancolitis). No toxicity of grade 4 was detected [129]. In a phase I/II study of monalizumab and durvalumab in solid tumors, safety, and elevated anti-tumor immune response were observed; however, the combination exhibited only modest efficacy [130] (Table 4).

There are currently 21 phase I and 36 phase II clinical trials registered on https://clinicaltrials.gov/ (accessed on 7 May 2025) that investigate durvalumab for CRC treatment.

### 4.6. Envafolimab

Envafolimab, or KN035, is a unique homodimeric fusion protein comprising a humanized single-domain PD-L1 antibody sourced from camels and a modified Fc region of human immunoglobulin G1, covalently connected by interchain disulfide bonds [131]. A pivotal phase II open-label study by Li et al. evaluated the efficacy and safety profile of envafolimab. The trial enrolled 103 participants across 25 centers in China with confirmed locally advanced or metastatic dMMR or MSI-H solid tumors. Participants received 150 mg of envafolimab via subcutaneous injection on a 28-day cycle. This subcutaneous route of administration offers a significant advantage over traditional intravenous delivery by reducing the risk of infusion-related adverse events, which is particularly beneficial in oncology settings. The study reported an ORR of 42.7% and a DCR of 66%, underscoring envafolimab’s favorable safety and efficacy profile. These outcomes are comparable to those observed in phase II studies of pembrolizumab and nivolumab [132].

Envafolimab has also shown potential as a neoadjuvant immunotherapy for locally advanced dMMR/MSI-H colon cancer. In a recent study conducted at Sun Yat-sen University Cancer Center and Yunnan Cancer Hospital, 15 patients receiving envafolimab were evaluated. The primary endpoint, pCR, was achieved in seven patients, while five others showed tumor regression, yielding a total CR rate of 66.7%. Clinical outcomes were promising, with six complete responses, five partial responses, and four cases of stable disease. The majority of treatment-related adverse events, such as pruritus and rash, were mild, and they occurred in 40% of patients. During a 7.9-month follow-up, no recurrences were observed, highlighting envafolimab’s safety and efficacy [133]. Table 5 summarizes the findings on envafolimab in CRC treatment.

There are currently 12 phase II trials and one phase III trial registered on https://clinicaltrials.gov/ (accessed on 7 May 2025) that investigate envafolimab for CRC treatment. The phase II/III RCT (NCT06959693) will evaluate the efficacy and safety of a combination of envafolimab, cetuximab-β, and mFOLFOX6 in patients with mCRC that is MSS and *RAS*/*BRAF* wild-type—a subgroup known to have limited response to immunotherapy alone. The trial will include patients with unresectable metastatic or recurrent colorectal adenocarcinoma who have not received prior systemic therapy for their metastatic disease. Participants will be randomly assigned to either the experimental group (receiving envafolimab + cetuximab-β + mFOLFOX6) or a control group receiving standard treatment.

### 4.7. Sintilimab

In preclinical studies, the combination of fruquintinib and sintilimab demonstrated efficient suppression of tumor growth and resulted in the elongation of survival span in mice with MC38 or CT26 xenograft tumors, as compared to the individual administration of fruquintinib or sintilimab. The combination of fruquintinib and sintilimab decreased the formation of new blood vessels, increased the presence of CD8+ T cells, CD8+ TNFα+ T cells, and CD8+ IFNγ+ T cells, and decreased the proportions of MDSCs and macrophages in mice. No apparent damage was seen in the primary organs of the mice following both types of treatment. In addition, the therapy utilizing the combination of fruquintinib and sintilimab demonstrated a successful response in five patients with treatment-resistant advanced MSS CRC [134].

The combination of fruquintinib and anti-PD-1 agent showed therapeutic efficacy in a small number of patients with extensively treated mCRC. Out of the 45 patients, the ORR was 11.1%, the DCR was 62.2%, the median PFS was 3.8 months, and the median OS was 14.9 months. The difference in PFS between the left and right primary tumors, as well as the difference in PFS in patients with or without lung metastases, did not reach statistical significance. There were no deaths reported that were attributed to adverse effects [135].

In a retrospective study, the efficacy and safety of combining regorafenib with anti-PD-1 antibodies have been demonstrated in patients with pMMR/MSS mCRC. This treatment has shown a tolerable safety profile and has been found to enhance the prognosis, particularly in patients who have undergone more than one treatment cycle. Sintilimab has been demonstrated to exhibit a significantly superior PFS in comparison to other anti-PD-1 antibodies, including nivolumab, pembrolizumab, camrelizumab, or toripalimab [136].

Following a series of six injections, each containing 200 mg of neoadjuvant anti-PD-1 therapy administered at three-week intervals, 90.9% (10 out of 11) of the patients with confirmed dMMR and MSI-H CRC successfully attained a pCR. The second patient, who experienced a significant pCR with less than 1% remaining tumor, had dMMR but MSS disease. There were no adverse effects connected to immunotherapy that reached a grade 3 severity or higher [137].

In a phase 1b/2 study where patients with advanced solid tumors or mCRC were given varying doses of fruquintinib along with a fixed dose of sintilimab, with the treatment administered either once every 4 weeks (Q4W) or once every 3 weeks (Q3W), a total of 23 patients were included in the dose-escalation group, and 37 patients were included in the mCRC cohort of the dose-expansion group. A combined analysis was conducted on 44 patients with mCRC who received sintilimab Q3W. During the dose escalation, there was one occurrence of a dose-limiting adverse event, namely, an elevation in troponin T. Grade ≥3 treatment-related adverse events were observed in 43.5% and 47.7% of patients during the dose-escalation phase and pooled analysis of mCRC, respectively. In the analysis of the combined data from patients with mCRC who were treated with the recommended dose of fruquintinib (5 mg once daily, 2 weeks on/1 week off) and sintilimab (200 mg every 3 weeks), the ORR was 23.8% The median PFS was 6.9 months, and the OS was 14.8 months. Among patients with pMMR mCRC, the ORR was 20.0%, the median PFS was 6.9 months, and the OS was 20.0 months [138].

As previously mentioned, the occurrence of mutations in the *RAS* gene is a frequent molecular event in CRC. The outlook for patients with mCRC who have an *RAS* mutation is unfavorable. One of the established treatment protocols for these patients as a first-line therapy is the combination of capecitabine and oxaliplatin (CapeOx), along with bevacizumab. This regimen has demonstrated an ORR of approximately 50% and a median PFS of 8–9 months. A phase II trial was conducted in China to evaluate the effectiveness of a treatment regimen consisting of intravenous administration of sintilimab (200 mg on day 1), along with bevacizumab (7.5 mg/kg on day 1), oxaliplatin (135 mg/m^2^ on day 1), and oral capecitabine (1 g/m^2^ on days 1–14) in a 21-day cycle in patients with unresectable, *RAS*-mutant, and MSS metastatic colorectal adenocarcinoma. Out of the 25 patients who participated in the trial, 2 (8%) patients experienced CR, 19 (76%) patients had PR, and 4 (16%) patients maintained SD. The ORR achieved a value of 84% Additionally, the DCR reached 100%. The entire group analyzed had a median PFS of 18.2 months. A combination therapy consisting of sintilimab, bevacizumab, oxaliplatin, and capecitabine has demonstrated notable efficacy in suppressing tumor development and maintaining an acceptable safety profile in patients with *RAS*-mutant, MSS, and unresectable mCRC, as a first treatment. The exploratory examination of biomarkers revealed that certain patients with *RAS* mutations and MSS status transitioned into an “immune-hot” category following the treatment [139].

Anlotinib is an oral tyrosine kinase inhibitor (TKI) that targets multiple receptors involved in tumor growth and angiogenesis, including VEGFR, FGFR, and PDGFR, and has shown promising efficacy and safety in the treatment of mCRC. Multiple studies have demonstrated that anlotinib administration significantly improves ORR and DCR in patients with mCRC. The investigation into the efficacy of the combination of sintilimab and anlotinib hydrochloride in the treatment of MSS CRC produced several noteworthy findings: (1) the observation group, which received the combination therapy, showed a considerably higher level of effectiveness in the short term compared to the control group (76.09% vs. 50%); (2) following treatment, the observation group showed notable reductions in blood levels of carcinoembryonic antigen (CEA), carbohydrate antigen 19-9 (CA19-9), and cancer antigen 125 (CA125), in comparison to the control group; (3) the levels of T-cell subsets in the observation group showed significant improvements after treatment, surpassing both the levels before treatment and the levels after treatment in the control group; (4) the observation group exhibited substantial enhancements in many aspects of QoL, surpassing both the initial levels before treatment and the levels after treatment in the control group; and (5) the observation group had a higher survival rate (73.33%) compared to the control group (52.27%) during the 1-year follow-up period, as shown by Kaplan–Meier survival analysis. Overall, the use of sintilimab and anlotinib hydrochloride exhibited promising effectiveness in managing MSS CRC patients, resulting in enhancements in patient immune response [140] (Table 6).

There are currently 7 phase I, 51 phase II, and 9 phase III trials on sintilimab and its combination with other drugs in CRC treatment registered on https://clinicaltrials.gov/ (accessed on 7 May 2025); the details of the phase III trials are summarized in Table 7.

### 4.8. Spartalizumab

Spartalizumab (PDR001) has undergone assessment in clinical trials for advanced solid tumors in conjunction with other antibodies, such as sabatolimab (TIM-3 inhibitor). A study conducted on recurrent/metastatic nasopharyngeal cancer found that spartalizumab exhibited a safety profile comparable to other anti-PD-1 antibodies. Moreover, it demonstrated a longer median OS and DoR, when compared to chemotherapy [141]. A phase I/II clinical trial assessed the safety and effectiveness of sabatolimab, either alone or in combination with spartalizumab, in patients diagnosed with advanced solid tumors. The main goals of phases I/Ib were to assess the safety and determine the recommended dose for phase II trials. Sabatolimab was given intravenously at doses ranging from 20 to 1200 mg, either every 2 weeks or every 4 weeks. Spartalizumab was administered intravenously at a dose of 80 to 400 mg, every 2 weeks or every 4 weeks. A total of 219 individuals were included in the study, with a variety of malignancies, including ovarian (17%) and CRC (7%). Among the enrolled patients, 133 received sabatolimab alone, while 86 received a combination of sabatolimab and spartalizumab. The combination of sabatolimab and spartalizumab demonstrated favorable tolerability and exhibited initial indications of anticancer efficacy. The RP2D for sabatolimab was determined to be 800 mg administered every 4 weeks (alternatively, every 3 weeks or every 2 weeks, based on modeling), with or without 400 mg of spartalizumab administered every 4 weeks [142].

There are currently 10 phase I trials and 3 phase II trials on spartalizumab and its combination with other drugs in CRC treatment registered on https://clinicaltrials.gov/ (accessed on 7 May 2025).

### 4.9. Tislelizumab

Tislelizumab is a monoclonal immunoglobulin G4 antibody that acts as an antagonist of the PD-1. Tislelizumab’s structure has been altered to effectively block the interaction between PD-1 and PD-L1, while reducing its affinity for Fcγ receptors. Tislelizumab has demonstrated initial anti-tumor efficacy in diverse solid tumors, either alone or in conjunction with ociperlimab (a humanized mAb that targets TIGIT and has shown promising results in cancer immunotherapy). Currently, it is being studied for its potential use in MSI-H/dMMR tumors, including CRC cancer [143].

In a single-arm, phase II trial, patients with mid-to-low locally advanced rectal cancer underwent long-course radiotherapy, consisting of 50 Gy delivered in 25 fractions at a rate of 2 Gy per fraction, over 5 days per week. Additionally, they received three cycles of capecitabine, administered at a dose of 1000 mg/m^2^ twice daily for 14 days, followed by three cycles of tislelizumab, given at a dose of 200 mg on day 8 of each 21-day cycle. Finally, the patients underwent radical surgery 6–8 weeks after completing the radiotherapy treatment. According to the interim findings, out of the 26 patients who underwent chemoradiotherapy, 24 patients were given three cycles of tislelizumab, while 2 patients received two treatments. Ultimately, 50% of patients with pMMR tumors achieved pathological full remission. A total of 19.2% of patients experienced an immune-related adverse event. Patients who did not have an elevated CEA level or were younger than 50 years old were more likely to get positive outcomes from this therapy regimen [144].

During the phase II trial, patients with MSS mCRC were given a combination of fecal microbiota transplantation (FMT), tislelizumab, and fruquintinib as a treatment option after two previous lines of therapy. The combination of FMT, tislelizumab, and fruquintinib enhanced survival outcomes in patients with refractory MSS mCRC. The median PFS was 9.6 months, the median OS was 13.7 months, the median DoR was 8.1 months, ORR was 20%, DCR was 95%, and CBR was 60%. Furthermore, the treatment exhibited a manageable safety profile. These findings suggest that this therapeutic approach presents a valuable new option for patients in this specific population [145] (Table 8).

There are currently 12 phase I, 66 phase II, and 7 phase III clinical trials registered at https://clinicaltrials.gov/ (accessed on 7 May 2025) that investigate tislelizumab for CRC treatment. The phase III trials are summarized in Table 9.

### 4.10. Toripalimab

Toripalimab is a selective, genetically engineered, humanized mAb that targets PD-1. It was created by Shanghai Junshi Bioscience Co., Ltd. (Shanghai; China). Conditional approval has been granted in China for the treatment of melanoma, nasopharyngeal carcinoma, and urothelial carcinoma. Additionally, the combination of axitinib with toripalimab has yielded considerable benefits in treating metastatic mucosal melanoma. Toripalimab has demonstrated favorable tolerability in patients and holds promise as a viable option for tumor treatment in the future [146,147,148].

The phase Ib/II clinical trial investigating regorafenib in combination with toripalimab in patients with mCRC reported several key findings: (1) efficacy—among patients treated with the RP2D (regorafenib 80 mg plus toripalimab), the combination achieved an ORR of 15.2% and a DCR of 36.4%, with median PFS and OS of 2.1 months and 15.5 months, respectively; (2) safety—the regimen was generally well tolerated, with treatment-related adverse events observed in 94.9% of patients (grade 1) and 38.5% (grade 3); (3) microbiome correlation—analysis revealed a negative association between treatment response and the relative abundance of *Fusobacterium* in the gut microbiome, suggesting a potential biomarker for therapeutic outcome [149].

Yu et al. assessed the effectiveness and safety of the combination of regorafenib and toripalimab in patients with recurrent or advanced CRC. The study comprised a cohort of 33 individuals diagnosed with advanced CRC, and the findings exhibited encouraging outcomes. The effectiveness of the treatment was assessed using the ORR, DCR, and PFS. The treatment responses were assessed independently, revealing that 12.12% of patients attained PR, 36.36% had SD, and 51.52% experienced progressive disease. The ORR was 12.12% and the DCR was 48.48%. The median PFS was 113 days. The predominant treatment-associated side effects included hand–foot syndrome, hepatic dysfunction, hypothyroidism, pyrexia, and weariness. The prevalence of severe (grade 3/4) adverse events was 9.09%. The study determined that the combination of regorafenib and toripalimab holds significant potential as a third-line treatment for patients with advanced CRC, namely, those who exhibit MSS. Nevertheless, additional investigations and extensive clinical trials are necessary to properly assess the effectiveness of this combination therapy in comparison with other conventional treatment choices for advanced CRC [150].

Nineteen patients with resistant or advanced MSS mCRC participated in a single-arm, single-center, prospective, phase II clinical study. They were administered fruquintinib orally at a dosage of 5 mg once daily for 3 weeks, followed by a 1-week break. This treatment cycle lasted for 4 weeks. Additionally, they received toripalimab intravenously at a dosage of 240 mg on day 1 once every 3 weeks. Treatment continued until disease progression or the occurrence of intolerable toxicity. The ORR was 21.05%. The median PFS and OS were 5.98 months and 11.1 months, respectively. Combining multiple therapies led to a longer PFS for individuals with peritoneal metastases. The most common side effects related to the drug were fatigue, liver malfunction, and high blood pressure. No substantial adverse effects or deaths related to adverse effects were observed. The study provided evidence demonstrating that the use of fruquintinib in combination with an anti-PD-1 monoclonal antibody is more efficacious, compared to fruquintinib alone, for treating Chinese patients with MSS advanced CRC in the third-line setting [151].

Hu et al. conducted a study to evaluate the efficacy and safety of toripalimab, administered either as monotherapy or in combination with the COX-2 inhibitor celecoxib, as neoadjuvant treatment for patients with dMMR/MSI-H CRC. In the combination group, 15 of 17 patients (88%) achieved a pCR, compared to 11 of 17 patients (65%) in the toripalimab monotherapy group. All patients subsequently received adjuvant toripalimab, with or without celecoxib, over a total perioperative period of six months. At the time of data cutoff, no recurrences were reported, and all patients were alive. The regimen was associated with a high pCR rate and a favorable safety profile. However, longer follow-up is required to assess survival-related outcomes [152] (Table 10).

There are currently 7 phase I and 26 phase II clinical trials registered at https://clinicaltrials.gov/ (accessed on 7 May 2025) that investigate toripalimab for CRC treatment.

Lynch Syndrome (LS) is the most common hereditary colorectal cancer syndrome, accounting for ~3% of all CRCs and increasing the lifetime risk of CRC and several extracolonic malignancies. Even after primary tumor resection, LS patients are at high risk for developing secondary primary tumors and precancerous adenomatous polyps. Given the proven efficacy of PD-1 inhibitors in dMMR (common in LS), the phase III study (NCT04711434) aims to assess whether immunoprevention with toripalimab can reduce future tumor formation.

## 5. Conclusions and Prospects

Targeting the PD-1/PD-L1 pathway represents a transformative approach in CRC treatment, showcasing the power of immunotherapy to harness the immune system against cancer. Monoclonal antibody-based inhibitors of this immune checkpoint have significantly advanced the field by effectively reactivating T-cell responses directed against the tumor. These therapies have achieved remarkable success in patients with MSI-H or dMMR CRC, in both of which the high neoantigen burden drives robust immunogenicity. While challenges remain in MSS CRC due to its less inflamed tumor microenvironment, ongoing research into combination therapies and mechanisms of resistance holds promise for expanding their efficacy. The advent of PD-1/PD-L1 inhibitors underscores a critical milestone in CRC management, offering improved outcomes and hope for broader therapeutic success as discussed below [153,154].

FDA-approved PD-1/PD-L1 inhibitors such as nivolumab, pembrolizumab, and dostarlimab have demonstrated efficacy in clinical trials, with established response criteria guiding their use. However, a range of other inhibitors, including AMP-224, atezolizumab, avelumab, camrelizumab, durvalumab, envafolimab, sintilimab, spartalizumab, tislelizumab, and toripalimab, remain under investigation for CRC treatment, showing potential but not yet achieving regulatory approval.

Despite these advancements, the application of PD-1/PD-L1 inhibitors in CRC faces significant challenges, particularly in tumors with pMMR or MSS phenotypes, a group which constitutes the majority of CRC cases. These tumors are characterized by a low TMB and limited immune infiltration, leading to reduced immunogenicity and poor responses to checkpoint blockade. Additionally, primary and acquired resistance mechanisms, such as alterations in antigen presentation machinery, upregulation of alternative immune checkpoints, and the immunosuppressive tumor microenvironment, further limit the efficacy of these therapies. While their use in dMMR or MSI-H CRC has been more successful, even these patients do not always achieve durable responses. Strategies to augment responses include combination therapies with other immunomodulators, such as CTLA-4 inhibitors, or targeted therapies like VEGF inhibitors, which may enhance immune cell infiltration and function. However, these approaches come with disadvantages, including increases in toxicity, cost, and the complexity of treatment regimens, necessitating careful patient selection.

**Figure 3:**
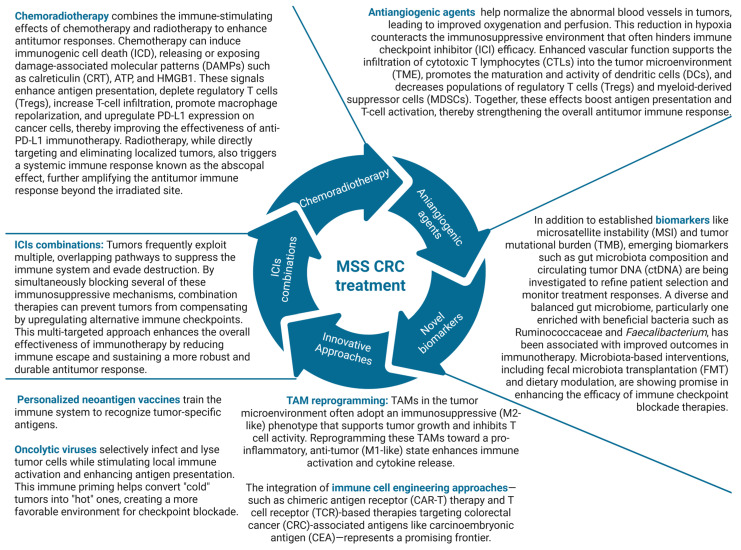
Strategies adapted to overcome the resistance to PD-1/PD-L1 checkpoint blockade therapies in MSS CRC. Created in BioRender. Kciuk, M. (2025) https://BioRender.com/l6u1uk5 (accessed on 20 June 2025).

Beyond MSI and TMB, new biomarkers, such as gut microbiota composition and ctDNA, are being explored to optimize patient selection and monitor therapy response. A diverse and balanced gut microbiome, enriched with beneficial bacteria like *Ruminococcaceae* and *Faecalibacterium*, has been linked to enhanced immunotherapy outcomes, while microbiota-based interventions, including fecal microbiota transplantation (FMT) and dietary modulation, offer promising avenues to improved checkpoint blockade efficacy [155,156,157]. Additionally, ctDNA serves as a non-invasive biomarker for real-time monitoring of tumor evolution, enabling early detection of resistance and personalized treatment adjustments, though challenges in sensitivity and specificity remain [158]. Innovations in personalized neoantigen vaccines, which train the immune system to recognize tumor-specific antigens, have demonstrated potential when combined with PD-1/PD-L1 inhibitors, particularly in refractory CRC subtypes [159]. Further, modulation of the TME through approaches such as oncolytic viruses, TAM reprogramming, and fibroblast-targeting therapies aims to convert immune-resistant “cold” tumors into inflamed “hot” tumors, thereby improving immunotherapy responsiveness [160]. The integration of immune cell engineering, including chimeric antigen receptor therapy (CAR-T) and TCR-therapies targeting CRC-associated antigens like CEA, represents another promising frontier, albeit one with challenges related to toxicity and limited efficacy in solid tumors [161]. Figure 3 illustrates the emerging strategies being utilized to overcome the resistance to PD-1/PD-L1 checkpoint blockade therapies in MSS CRC (Figure 3).

Emerging applications of artificial intelligence (AI) in medical imaging and biomarker analysis hold potential for refining patient stratification and predicting immunotherapy responses with greater precision, though regulatory and ethical challenges must be addressed before widespread clinical adoption. As these advancements continue to evolve, a multi-faceted approach combining biomarker identification, TME modulation, immune engineering, and AI-driven precision medicine may ultimately enhance the effectiveness of PD-1/PD-L1 inhibitors in CRC, expanding their applicability beyond MSI-H/dMMR tumors and reshaping the future of immunotherapy with respect to this malignancy [162,163].

## Figures and Tables

**Table 1:** Registered phase III clinical trials on atezolizumab for colorectal cancer (CRC) treatment (source: https://clinicaltrials.gov/). The table excludes completed, suspended, terminated and unknown status studies.

Number	Short Description and Rationale	Status	Estimated Enrollment
NCT05770102	This clinical trial is evaluating the effectiveness of atezolizumab in treating rare or less common cancer types that exhibit high TMB, MSI-H, or constitutional dMMR. Atezolizumab is already approved in the UK for several cancers, including urothelial cancer, non-small cell lung cancer, and triple-negative breast cancer. The aim of this study is to determine whether the drug could also benefit patients with other cancers that share similar genetic features. If successful, the findings may support broader National Health Service (NHS) access to atezolizumab through the Cancer Drugs Fund. This trial is part of the larger DETERMINE programme, which is exploring targeted treatments for rare cancers based on genetic markers.	Recruiting	30
NCT05425940	This study aims to compare the effects of a combination of XL092 and atezolizumab versus regorafenib in patients with MSS/MSI-low mCRC. Eligible participants are those whose cancer has progressed during or after, or who are intolerant to, standard-of-care treatments. The rationale for combining XL092, a next-generation tyrosine kinase inhibitor, with atezolizumab, an immune checkpoint inhibitor, is based on the potential for synergistic effects. XL092 may help reshape the TME and reduce immune suppression, potentially enhancing the anti-tumor immune response triggered by atezolizumab. This combination may offer improved outcomes compared to regorafenib, a standard treatment option for refractory mCRC.	Active, not recruiting	874
NCT05141721	This clinical trial is evaluating a personalized cancer vaccine approach in combination with standard therapy for patients with advanced solid tumors. The study consists of two phases. In Phase 2, the goal is to assess the biological activity of patient-specific vaccines (GRT-C901 and GRT-R902) combined with ICIs and standard maintenance therapy (fluoropyrimidine and bevacizumab), compared to standard therapy alone. The primary measure of response is a reduction in ctDNA. In Phase 3, the study aims to determine the clinical effectiveness of the combination regimen by measuring PFS. The personalized vaccines are designed based on mutations unique to each patient’s tumor. These mutations can produce neoantigens that trigger an immune response when presented on the tumor cell surface. The vaccine uses a heterologous prime-boost strategy (first GRT-C901, then GRT-R902) to enhance T-cell activation against these neoantigens and support the efficacy.	Active, not recruiting	700
NCT06733038	This trial is investigating whether adding atezolizumab to standard first-line chemotherapy (FOLFOXIRI plus bevacizumab) improves outcomes in patients with pMMR mCRC who are classified as immunoscore high. Patients will be assigned to one of two treatment groups: Arm A (control), FOLFOXIRI plus bevacizumab for up to 8 cycles, followed by maintenance therapy with 5-FU/leucovorin plus bevacizumab. Arm B (experimental), FOLFOXIRI plus bevacizumab and atezolizumab for up to 8 cycles, followed by maintenance therapy with 5-FU/leucovorin, bevacizumab, and atezolizumab. The primary goal is to determine whether the addition of atezolizumab extends PFS.	Recruiting	238
NCT05482516	This pilot feasibility study is evaluating the use of atezolizumab and bevacizumab as adjuvant immunotherapy in patients with gastrointestinal cancers who have no evidence of disease on imaging but show minimal residual disease based on a positive Signatera™ ctDNA test. Although standard imaging may show no detectable disease after treatment, the presence of tumor DNA in the bloodstream (positive ctDNA) suggests a high risk of relapse. This study explores whether early intervention with immunotherapy (atezolizumab) and anti-angiogenic therapy (bevacizumab) can prevent or delay recurrence by targeting microscopic residual disease before it becomes clinically evident. All participants must have completed standard-of-care, curative-intent treatment (e.g., surgery, chemotherapy, radiation) and be enrolled within one year of treatment completion. Eligible patients will receive intravenous atezolizumab (1200 mg) and bevacizumab (15 mg/kg) every 21 days, continuing until disease recurrence, progression on ctDNA (molecular relapse), unacceptable toxicity, withdrawal of consent, or up to a maximum of 12 months.	Recruiting	20
NCT04157985	PD-1/PD-L1 inhibitors have shown significant benefit in treating various cancers, but the ideal treatment duration remains unknown. Prolonged therapy may expose patients to unnecessary side effects and healthcare costs. This clinical trial aims to determine the optimal duration of PD-1/PD-L1 immunotherapy in patients with advanced solid tumors who have achieved stable disease. Patients who have been on treatment for one year and have no disease progression will be randomized to either stop therapy or continue until disease progression. The trial is being conducted within the University of Pittsburgh Medical Center (UPMC) health system, where over 2300 patients received PD-1/PD-L1 inhibitors in the past year for a range of advanced cancers. The study was initiated in response to a survey of oncologists within the system, the vast majority of whom expressed strong interest in participating in research to evaluate whether treatment can safely be stopped after one year.	Recruiting	578
NCT02912559	dMMR tumors tend to produce more neoantigens, making them more visible to the immune system. This suggests that immunotherapy may be especially effective in this group. By combining atezolizumab with chemotherapy, the study aims to determine if this approach can reduce the risk of cancer recurrence and improve survival compared to chemotherapy alone. Participants will be randomly assigned to receive either standard chemotherapy (oxaliplatin, leucovorin calcium, and fluorouracil) alone or in combination with atezolizumab.	Active, not recruiting	700

**Table 2:** Summary of clinical trials investigating avelumab efficiency and safety in metastatic colorectal cancer.

NCT Number	Phase	Treatment	Findings	PMID
NCT01772004	Phase I	Avelumab	No objective responses; median PFS of 2.1 months; five grade 3 TRAEs.	37310790
NCT03152565	Phase I/II	Avelumab plus autologous dendritic cell (ADC) vaccine	Combined therapy safe and well-tolerated; 11% of patients disease-free at 6 months; median PFS of 3.1 months; metabolic rewiring noted post-therapy.	36083313
NCT03186326	Phase II	Avelumab vs. standard second-line chemotherapy	Avelumab superior to chemotherapy in PFS for dMMR/MSI mCRC; fewer grade 3+ TRAEs; better disease control duration with avelumab.	37535388

**Table 3:** Summary of clinical trials investigating camrelizumab efficiency and safety in colorectal cancer (CRC).

NCT Number	Phase	Treatment	Findings	PMID
NCT04231552	II	Preoperative short-course radiotherapy, CAPOX (capecitabine and oxaliplatin), and camrelizumab	pCR rate of 48.1% (13/27). Grade 1–2 AEs; no grade 4/5 AEs. Better pCR tendency without *FGFR1-3* deletions.	34725214
N/A (Retrospective Study)	N/A	Camrelizumab, XELOX (capecitabine and oxaliplatin), and bevacizumab or regorafenib	ORR 72%, DCR 96%. Median PFS 11.2 months. Most AEs were grade 1 or 2; grade 3 toxicities occurred in 32% of patients.	34900725
N/A (Cohort B of CRACK Study)	II	Cetuximab, camrelizumab, and liposomal irinotecan	ORR 25%, DCR 75%. Median PFS 6.9 months, median OS 15.1 months. Grade 3 TRAEs in 15.8% of patients, no grade ≥4 TRAEs.	37163613

**Table 4:** Summary of clinical trials evaluating immunotherapy combinations with durvalumab in colorectal cancer (CRC).

NCT Number	Phase	Treatment	Findings	PMID
NCT03122509	Phase II	Durvalumab combined with tremelimumab and radiotherapy	ORR 8.3%, median PFS 1.8 months, median OS 11.4 months, treatment-related grade 3–4 AEs in 25%	33504552
NCT02754856	Phase II	Durvalumab combined with tremelimumab (perioperative)	74% underwent resection, RFS 9.7 months, OS 24.5 months, 4 complete pCRs	33811152
NCT04083365	Phase II	Durvalumab (monotherapy)	ORR 42.4%, 12-month PFS 58.2%, 12-month OS 68.3%, 36.4% with grade 3 AEs	35179785
NCT03206073	Phase I/II	Durvalumab combined with PexaVec with/without tremelimumab	Median PFS 2.3 months, no unexpected toxicities, increased CD8+ T-cell activation	36754451
NCT03202758	Phase 1b/2	Durvalumab combined with tremelimumab and mFOLFOX6	3-month PFS 90.7%, ORR 64.5%, median PFS 8.2 months, promising clinical activity in MSS mCRC	37563240
NCT04083365	Phase II	Durvalumab (neoadjuvant) plus capecitabine-based chemoradiotherapy	pCR 34.5%, safe toxicity profile, promising neoadjuvant strategy	37774508
NCT02671435	Phase 1/2	Durvalumab and monalizumab	Modest efficacy (7.7% MSS-CRC response), immune activation observed in TME	38309722

**Table 5:** Clinical data on envafolimab efficiency and safety in colorectal cancer (CRC).

NCT Number	Phase	Treatment	Findings	PMID
NCT03667170	Phase 2	Subcutaneous envafolimab monotherapy	ORR: 42.7%; DCR: 66.0%; median PFS: 11.1 months; OS at 12 months: 74.6%.	34154614
Not provided	Not provided	Neoadjuvant subcutaneous envafolimab	66.7% pCR rate. Most common AEs: pruritus and rash (40%). No recurrences at 7.9 months follow-up.	38691294

**Table 6:** Clinical data on sintilimab efficiency and safety in colorectal cancer (CRC).

NCT Number	Phase	Treatment	Findings	PMID
NCT03903705	Phase 1b/2	Fruquintinib plus sintilimab in advanced solid tumors and metastatic colorectal cancer (mCRC).	ORR: 23.8%, median PFS: 6.9 months, median OS: 14.8 months. Grade ≥3 TRAEs: 47.7%.	36628898
N/A	N/A	Fruquintinib plus PD-1 inhibitors in refractory non-MSI-H/pMMR mCRC (real-world study).	ORR: 11.1%, DCR: 62.2%, median PFS: 3.8 months, median OS: 14.9 months. No adverse-effect-related deaths.	35875064
N/A	N/A	Anti-PD-1 antibody plus regorafenib in refractory pMMR/MSS mCRC (retrospective cohort study).	ORR: 12.7%, DCR: 41.8%, median OS: 8.4 months, median PFS: 2.5 months. Grade ≥3 TRAEs: 12.6%.	36111036
NCT04194359	Phase II	Sintilimab plus bevacizumab, oxaliplatin, and capecitabine in RAS-mutant, MSS, unresectable mCRC.	ORR: 84%, DCR: 100%, median PFS: 18.2 months. No grade 5 TRAEs.	37554125
N/A	N/A	Sintilimab combined with anlotinib hydrochloride in MSS CRC treatment (comparative analysis).	ORR: 76.09%, improved quality of life, survival rate: 73.33%. Comparable safety profile.	38077647
N/A	N/A	Single-agent neoadjuvant PD-1 antibody (sintilimab) in locally advanced dMMR/MSI-H CRC.	pCR in 90.9%, no grade 3 or above immunotherapy-related adverse events.	36528470

**Table 7:** Registered phase III clinical trials on sintilimab for colorectal cancer (CRC) treatment (source: https://clinicaltrials.gov/).

Number	Short Description and Rationale	Status	Estimated Enrollment
NCT05236972	This open-label Phase III clinical trial will compare the effectiveness of sintilimab alone versus standard chemotherapy (XELOX) in patients with locally advanced, dMMR or MSI-H CRC. Eligible patients must have no distant metastases (M0), lymph node involvement (N+), and tumors located at least 10 cm from the anal verge. Participants will be randomized into two treatment groups: Group A (Immunotherapy arm): Anti-PD-1 antibody (200 mg IV every 3 weeks) for 8 cycles. Group B (Chemotherapy arm): XELOX regimen (oxaliplatin + capecitabine) for 4 or 8 cycles, repeated every 21 days. The primary endpoint is 3-year DFS, assessed in all patients with post-randomization data.	Recruiting	323
NCT06497985	MSS/pMMR colorectal cancers are typically resistant to immunotherapy alone. This study combines epigenetic modulation (tucidinostat), immune checkpoint inhibition (sintilimab), and anti-angiogenic therapy (bevacizumab) in an effort to sensitize these tumors to immune attack. The control, fruquintinib, represents the current standard for treatment-refractory MSS mCRC. This trial aims to determine whether the combination approach improves survival outcomes compared to existing therapies. A total of 430 patients will be enrolled and randomized in a 1:1 ratio to: Experimental arm: tucidinostat (a histone deacetylase inhibitor) + sintilimab (a PD-1 inhibitor) + bevacizumab (an anti-VEGF antibody); Control arm: fruquintinib monotherapy (a VEGFR tyrosine kinase inhibitor approved for refractory mCRC).	Recruiting	430
NCT05171660	*RAS*-mutant mCRC patients typically do not benefit from anti-EGFR therapies and have limited targeted treatment options. This trial aims to determine whether the combination of immunotherapy and chemotherapy can improve clinical outcomes in this molecularly defined population, addressing an important unmet need in first-line mCRC treatment. This trial is evaluating the efficacy and safety of sintilimab in combination with XELOX (capecitabine + oxaliplatin) and bevacizumab as a first-line treatment for patients with *RAS*-mutant mCRC who have not received prior systemic therapy.	Recruiting	436
NCT06794086	SBRT precisely targets liver metastases with high-dose radiation, potentially increasing tumor antigen release and enhancing immune recognition. When combined with PD-1 blockade, this local-regional approach may amplify systemic anti-tumor immune responses, offering a promising strategy for otherwise inoperable liver metastases. This trial will evaluate the efficacy and safety of combining SBRT with a PD-1 monoclonal antibody for patients with unresectable colorectal cancer liver metastases. Eligible participants are those whose liver metastases are deemed unresectable by a multidisciplinary hepatobiliary team but are [found] suitable for SBRT by a radiation oncology team. All patients will receive hypofractionated SBRT (8–12 Gy over 5 fractions) alongside systemic therapy consisting of 5–FU–based chemotherapy and PD-1 immunotherapy, administered before and after radiotherapy.	Recruiting	24
NCT06791512	Standard immunotherapy has shown limited efficacy in pMMR/MSS CRC. However, preliminary data (from the earlier BASKET II study) suggest that combining chemotherapy and anti-angiogenic therapy with PD-1 blockade can enhance tumor immunogenicity, increase pCR rates, and improve the chance of R0 resection—a critical factor for long-term survival. This trial seeks to validate those findings on a larger scale and with longer-term endpoints. This RCT will evaluate the efficacy and safety of adding bevacizumab and a sintilimab to standard mFOLFOX6 neoadjuvant chemotherapy in patients with locally advanced pMMR/MSS CRC.	Recruiting	122
NCT05890742	MSI-H/dMMR colon cancers are highly immunogenic and respond well to ICIs. While PD-1 blockade alone has shown promise, combining it with CTLA-4 inhibition may further enhance anti-tumor immunity by promoting a broader and more robust T-cell response. The goal of this combination is to maximize tumor shrinkage prior to surgery. This prospective clinical trial is evaluating the efficacy and safety of IBI310, a CTLA-4 monoclonal antibody, in combination with sintilimab, a PD-1 inhibitor, as neoadjuvant therapy for patients with MSI-H/dMMR resectable colon cancer.	Recruiting	360
NCT05484024	Previous evidence from the STELLAR study demonstrated that short-course radiotherapy followed by chemotherapy (e.g., CAPOX) is non-inferior to traditional long-course chemoradiotherapy for rectal cancer. The combination of short-course radiotherapy and chemotherapy (CAPOX/mFOLFOX) may increase tumor immunogenicity, potentially making tumors more responsive to sintilimab.	Not yet recruiting	588
NCT05768503	The trial evaluates the efficacy and safety of a novel combination—chidamide (a histone deacetylase inhibitor), sintilimab (a PD-1 inhibitor), and bevacizumab (an anti-VEGF monoclonal antibody)—compared with the standard second-line regimen of FOLFIRI plus bevacizumab in patients with MSS mCRC who have progressed after first-line oxaliplatin-based therapy. Patients with MSS colorectal cancer tend to respond poorly to immunotherapy alone due to a relatively “cold” tumor microenvironment with low immune infiltration. The study combines chidamide, which modulates gene expression and may increase tumor immunogenicity by enhancing antigen presentation and reversing immune suppression, sintilimab, a PD-1 inhibitor that restores T-cell anti-tumor activity, and bevacizumab, which normalizes tumor vasculature and can promote immune cell infiltration. Together, these agents may synergize to overcome immune resistance in MSS CRC, offering an immunomodulatory alternative to conventional chemotherapy.	Recruiting	176
NCT05374252	Standard treatment for locally advanced anal canal squamous carcinoma involves mitomycin C + 5-FU chemotherapy combined with long-course IMRT. While this approach achieves reasonable local control, recurrence and distant metastasis remain challenges. Adding sintilimab, a PD-1 immune checkpoint inhibitor, may enhance anti-tumor immune responses during chemoradiation; improve tumor clearance, particularly in micrometastatic disease; and prolong PFS and OS compared to chemoradiotherapy alone. This trial will evaluate the efficacy and safety of adding the sintilimab to standard concurrent chemoradiotherapy in patients with locally advanced squamous cell carcinoma of the anal canal.	Recruiting	102

**Table 8:** Clinical data on tislelizumab efficiency and safety in colorectal cancer (CRC).

NCT Number	Phase	Treatment	Findings	PMID
NCT04911517	II	Long-course chemoradiotherapy combined with concurrent tislelizumab	Pathological complete remission was achieved in 50% (13/26) of patients; immune-related AEs occurred in 19.2% (5/26) of patients; favorable safety and efficacy; did not increase surgical complication rate.	36816939
Not provided (ChiCTR2100046768)	II	Fecal microbiota transplantation (FMT) plus tislelizumab and fruquintinib	Median PFS: 9.6 months; Median OS: 13.7 months; ORR: 20%; DCR: 95%; CBR: 60%; 95% experienced TRAEs; 30% had grade 3–4 TRAEs; high abundance of *Proteobacteria* and *Lachnospiraceae* linked to response; manageable safety profile.	38024475

**Table 9:** Registered phase III clinical trials on tislelizumab for colorectal cancer (CRC) treatment (source: https://clinicaltrials.gov/).

Number	Short Description and Rationale	Status	Estimated Enrollment
NCT06520683	Stage II dMMR/MSI-H CRC typically has a favorable prognosis, but optimal adjuvant treatment is not well defined. Neoadjuvant immunotherapy trials (e.g., NICHE-2) showed exceptionally low recurrence rates with only two cycles of PD-1 blockade, suggesting short-course immunotherapy could be sufficient. A short, low-toxicity regimen may improve long-term outcomes without exposing patients to unnecessary side effects. This trial was designed to assess the efficacy and safety of two cycles of adjuvant tislelizumab compared to standard-of-care in patients with stage II dMMR)/MSI-H CRC.	Recruiting	180
NCT06332274	MRD, detected via ctDNA, signals minimal disease presence even when imaging is clear. MRD+ patients have a significantly higher risk of relapse than those who are MRD negative. Given the efficacy of immunotherapy in advanced disease, there is strong interest in applying it earlier in the disease course, particularly in MRD+ settings. This is a biomarker-driven, single-arm clinical trial (UMBRELLA) evaluating the efficacy of tislelizumab in patients with solid tumors who are MRD+ after completing surgery and standard perioperative treatments.	Not yet recruiting	717
NCT06312982	Neoadjuvant chemoradiotherapy is the standard approach for locally advanced rectal cancer. Adding immune checkpoint inhibitors like tirelizumab may enhance tumor response and improve long-term outcomes, including sphincter preservation and quality of life. This trial will evaluate the efficacy and safety of adding tirelizumab (tislelizumab) to standard neoadjuvant chemoradiotherapy in patients with locally advanced rectal cancer.	Recruiting	375
NCT06017583	This is a phase III RCT evaluating the efficacy and safety of combining tislelizumab with SIB-IMRT and chemotherapy (capecitabine/XELOX) in patients with locally advanced rectal cancer.	Recruiting	48
NCT06443671	This is a prospective RCT evaluating the efficacy and safety of neoadjuvant fruquintinib and tislelizumab combined with mCapeOX versus CapeOX alone in patients with mid-high rectal cancer that is pMMR/MSS and locally advanced.	Not yet recruiting	132
NCT06507371	Standard radiotherapy for rectal cancer often includes tumor-draining lymph nodes, which may impair local immunity and increase toxicity. Node-sparing radiotherapy targets only the tumor bed, aiming to preserve immune function and minimize side effects. Combining this approach with CAPOX (capecitabine + oxaliplatin) and tislelizumab may improve tumor response by enhancing local and systemic anti-tumor immunity in MSS patients, who typically respond poorly to immunotherapy alone.	Recruiting	170

**Table 10:** Clinical data on toripalimab efficiency and safety in colorectal cancer (CRC).

NCT Number	Phase	Treatment	Findings	PMID
NCT03926338	Phase II	Neoadjuvant PD-1 blockade with toripalimab, with or without celecoxib, in mismatch repair-deficient or microsatellite instability-high colorectal cancer	pCR response: 88% with toripalimab + celecoxib, 65% with toripalimab alone. No treatment-related surgical delays. High pCR rate and acceptable safety profile.	34688374
Not available	Phase Ib/II	Regorafenib plus toripalimab in metastatic colorectal cancer	ORR: 15.2%, DCR: 36.4%, median PFS: 2.1 months, median OS: 15.5 months. Patients with liver metastases had lower ORR. High-abundance *Fusobacterium* linked to shorter PFS.	34622226
Not available	Phase II	Regorafenib combined with toripalimab in third-line-and-beyond treatment of advanced colorectal cancer	ORR: 12.12%, DCR: 48.48%, median PFS: 113 days. TRAEs included hand–foot syndrome (33.33%) and liver dysfunction (27.27%).	34603452
Not available	Phase II	Toripalimab with fruquintinib in refractory advanced metastatic colorectal cancer	ORR: 21.05%, median PFS: 5.98 months, median OS: 11.10 months. Peritoneal metastasis was associated with longer PFS. Common AEs: fatigue (57.89%), hepatic dysfunction (42.11%), hypertension (36.84%).	37201046

## Data Availability

No data was generated.

## Abbreviations

The following abbreviations are used in this manuscript:

ADC	Autologous dendritic cells
ADCC	Antibody-dependent cell-mediated cytotoxicity
BRAF	Serine/threonine-protein kinase B-Raf
CA125	Cancer antigen 125
CA19-9	Carbohydrate antigen 19-9
CAE	Carcinoembryonic antigen
CAR-T	Chimeric antigen receptor therapy
CBR	Clinical benefit rate
CCR2/5	C-C motif chemokine receptor 2/5
CD39/73	Cluster of differentiation
CEA	Carcinoembryonic antigen
CMS	Consensus molecular subtypes
COX-2	Cyclooxygenase-2
CPS	Combined positive score
CR	Complete response
CRC	Colorectal cancer
CtDNA	Circulating tumor DNA
CTLA4	Cytotoxic T-lymphocyte-associated protein 4
CTLA-4	Cytotoxic T-cell antigen 4
CTLs	Cytotoxic T cells
DCR	Disease control rate
dMMR	Mismatch repair-deficient
DoR	Duration of response
EGFR	Epidermal growth factor receptor
EORTC QLQ-C30	The European Organisation for Research and Treatment of Cancer Quality of Life Questionnaire Core 30
Fc	Fragment crystallizable
FGFR	Fibroblast growth factor receptor
FMT	Fecal microbiota transplantation
GITR	Glucocorticoid-induced TNFR-related
HIFs	Hypoxia-inducible factors
HMGB1	High mobility group box 1
HPV	Human papillomavirus
ICIs	Immune-checkpoint inhibitors
IFN-γ	Interferon gamma
IHC	Immunohistochemistry
IL	Interleukin
IND	Investigational new drug
irORR	Immune-related objective response rate
JNK	Janus kinase
KRAS	Kirsten rat sarcoma viral oncogene homolog
LAG3	Lymphocyte-activation gene 3
mCRC	Metastatic colorectal cancer
MDSC	Myeloid-derived suppressor cells
MEK	Mitogen-activated protein kinase kinase
MGMT	Methylguanine methyltransferase
MHC	Major histocompatibility complex
MLH1	MutL homolog 1
MSH2	MutS homolog 2
MSH6	MutS homolog 6
MSI-H	Microsatellite instability-high
NK	Natural killer
NTRK	Neurotrophic tropomyosin receptor kinases
ORR	Objective response rate
OS	Overall survival
PARP	Poly (ADP-ribose) polymerase
PBMCs	Peripheral blood mononuclear cells
pCR	Pathological complete response
PCR	Polymerase chain reaction
PD-1	Programmed death-1
PDGFR	Platelet-derived growth factor receptor
PD-L1	Programmed death ligand-1
PFS	Progression-free survival
pMMR	Proficient MMR
PMS2	Postmeiotic segregation increased 2
POLE EDM	Polymerase ε (POLE) exonuclease domain
PR	Partial response
PVRIG	PVR-related immunoglobulin domain containing protein; CD112R
QoL	Quality of life
RCTs	Randomized controlled trials
RP2D	Recommended phase II dosage
SBRT	Stereotactic body radiation treatment
SCCA	Squamous cell carcinoma of the anal canal
SD	Stable disease
SHP2	SRC homology 2 domain-containing phosphatase 2
STAT	Signal transducer and activator of transcription
TAMs	Tumor-associated macrophages
TCR	T-cell receptor
TFD	Trifluridine
TGF-β	Transforming growth factor beta
TIGIT	Anti-T-cell immunoglobulin and immunoreceptor tyrosine-based inhibitory motif domain
TILs	Tumor-infiltrating lymphocytes
TIM-3	T-cell immunoglobulin and mucin-domain containing-3
TKI	Tyrosine kinase inhibitor
TMB	Tumor mutational burden
TME	Tumor microenvironment
TMZ	Temozolomide
TNF-α	Tumor necrosis factor alpha
TPI	Tipiraci
T-reg	Regulatory T cell
VEGF	Vascular endothelial growth factor
VEGFR1-3	Vascular endothelial growth factor receptors 1-3
